# Nicotine-Induced VEGF Levels in NSCLC Cells Are Modulated by PKA, Hyaluronan, and p53

**DOI:** 10.3390/ijms262211103

**Published:** 2025-11-17

**Authors:** Caroline Wozniak, Alvaro Cobos, Aya Sabri, Stuti Goel, Brooke Lopo, Sarah Sarofim, Chanidapa Chutipassakul, Jeffrey Guthrie, Deborah Heyl, Hedeel Guy Evans

**Affiliations:** Chemistry Department, Eastern Michigan University, Ypsilanti, MI 48197, USA; cwoznia5@emich.edu (C.W.); acobos2@emich.edu (A.C.); asabri@emich.edu (A.S.); sgoel1@emich.edu (S.G.); blopo@emich.edu (B.L.); sjosep16@emich.edu (S.S.); cchutipa@emich.edu (C.C.); jguthri7@emich.edu (J.G.); dheylcle@emich.edu (D.H.)

**Keywords:** NSCLC, VEGF, nicotine, protein kinase A (PKA), hyaluronan (HA), p53, α-bungarotoxin, propranolol, caspase-3, extracellular

## Abstract

Nicotine promotes non-small cell lung cancer (NSCLC) survival in part by elevating vascular endothelial growth factor (VEGF), yet the upstream regulatory mechanisms remain unclear. Here we identify a PKA–HA–p53 regulatory axis that governs nicotine-driven VEGF levels and survival in A549 (p53^+^/^+^) and H1299 (p53-null) cells. Nicotine increased VEGF levels in the media, an effect augmented by protein kinase A (PKA) activation and reduced by PKA inhibition. Blocking hyaluronan (HA) synthesis with 4-methylumbelliferone (4-MU) lowered VEGF levels and diminished the nicotine response, suggesting that HA–CD44 contributes to PKA-linked survival pathways. In A549, p53 inhibition or knockdown enhanced PKA activity and VEGF levels, indicating that p53 constrains this axis; by contrast, H1299 displayed sustained nicotine responsiveness consistent with p53 loss. Pharmacologic nAChR/β-adrenergic blockade blunted nicotine-induced PKA signaling. Functionally, VEGF immunodepletion or co-treatment with a PKA inhibitor, 4-MU, or anti-VEGF antibodies reduced nicotine-supported viability and increased apoptosis, while the addition of purified VEGF rescued survival, establishing the role of VEGF in this pathway. Collectively, these findings delineate a mechanistic network in which PKA, HA–CD44 signaling, and p53 integrate nicotinic cues to control VEGF media levels and cell survival, identifying potential targets (PKA, HA synthesis, VEGF) for mitigating nicotine-mediated NSCLC progression.

## 1. Introduction

The incidence of the potentially fatal non-small cell lung cancer (NSCLC) continues to increase annually, highlighting the need for better understanding of the underlying molecular mechanisms that promote NSCLC cell growth [1,2]. NSCLC is the most common type of lung cancer, mainly composed of adenocarcinomas, and considered to be highly resistant to current cancer therapeutics [2].

Cigarette smoking is known to be a cause for NSCLC development, and nicotine has been reported to increase growth of cultured NSCLC cells [3,4,5,6,7]. Nicotine can accelerate many steps in cancer development and can facilitate mitogenic signaling pathways promoting cell proliferation and survival [4,7,8]. Nicotinic receptors have been reported in a variety of cancer cells to stimulate pro-mitotic and anti-apoptotic signaling cascades [6,7,8,9]. Nicotinic acetylcholine receptors (nAChRs) are known to mediate neurotransmission; however, several homomeric nAChRs composed of α-subunits and heteromeric nAChRs composed of both α- and β-subunits have been found in non-neuronal mammalian cells, regulating a variety of cellular functions [6,7,8,9,10]. Nicotine and acetylcholine (ACh) bind the nAChRs as the exogenous and endogenous ligand, respectively [7,10,11]. Multiple nAChR subunits are expressed in human NSCLC cell lines that include A549 and H1299 used in this study [12,13] with α7nAChR considered as the most growth stimulatory receptor that facilitates nicotine-mediated cell proliferation [6,7,14,15]. Nicotine is known to mediate its pathophysiological effects primarily via the α7-nAChRs, known to be overexpressed in NSCLC human tumors compared to normal tissue [5,10,14,16,17,18].

Nicotine stimulates pro-angiogenic signaling in NSCLC [6,8,19]. It upregulates hypoxia-inducible factor 1 α (HIF-1α) and Vascular Endothelial Growth Factor (VEGF) in human lung cancer cells (including A549), and many of these effects are mediated by α7-nAChR [5,20,21]. Adrenaline and noradrenaline have been shown in different cancer cell lines to act via β-adrenergic receptor (β-AR) signaling leading to elevated secreted levels of VEGF facilitating tumor growth [22], effects blocked by cell treatment with the β-AR antagonist, propranolol [22,23,24]. β-ARs are expressed in lung cancer cells and have been associated with increased cellular proliferation, metastasis, and reduced apoptosis [25]. Using A549 (p53 wild-type) and H1299 (p53-null) lung cancer cells, we reported that cell treatment with nicotine increased adrenaline and noradrenaline levels, an effect blocked by cell treatment with the α7nAChR inhibitor, α-bungarotoxin (α-BTX), but not by the β-blocker, propranolol [21]. These findings are consistent with studies using colon cancer cells which showed that binding of nicotine to α7nAChR increased noradrenaline levels, indirectly activating the β-AR signaling pathway [26,27]. Adrenaline and noradrenaline acting via β-AR in a variety of cancer cell types, including lung cancer, have been reported to have a strong growth stimulating effect [25,28,29,30,31,32]. Cell incubation with the β1/2-AR agonist, isoproterenol, was reported to increase expression of VEGF and matrix metalloproteinase-2/9 enhancing A549 cell proliferation [33].

Previously, we reported that nAChR, tropomyosin receptor kinase B TrkB, and β-AR, lead to regulation of epidermal growth factor receptor (EGFR) and the insulin-like growth factor 1 receptor (IGF-1R) in NSCLC, affecting PI3K/AKT signaling and chemoresistance [34]. We also showed that A549 cell treatment with nicotine, epinephrine, or brain-derived neurotrophic factor (BDNF) increased the levels of MMP9 and soluble E-cadherin (sE-cad) in the media, increasing cell survival and blocking apoptosis [35]. Moreover, we found that nicotine treatment of NSCLC cells upregulated the levels of VEGF and cell survival via α7nAChR and/or β-Ars, while GABA and/or p53 led to downregulation of VEGF levels [21]. While we previously reported that nicotine enhances VEGF signaling in NSCLC by acting positively via the α7nAChR and β-Ars, leading to increased cell survival and decreased apoptosis [21], more recently, we found that lactoferrin-induced activation of caspase-3 increased by inhibiting the function of VEGF in A549 and H1299 cells [36]. The glycosaminoglycan, hyaluronan (HA), is a non-sulfated [37,38,39,40] polymer that consists of the disaccharide sequence (D-glucuronic acid and D-N-acetylglucosamine) [40,41,42]. HA is a major component of the extracellular matrix (ECM) that activates signaling pathways promoting tumor progression by interactions with its major cell surface receptor, CD44 [38,39,41,43,44,45,46]. Tumor-suppressor functions of p53 are counteracted by high CD44 expression, while p53 acts to repress CD44 expression to promote its antiproliferative activities [47,48]. While minimal levels of HA have been reported in normal tissue, HA has been recently shown to accumulate in the tumor parenchyma in certain types of cancer, including lung cancer, contributing to increased tumorigenesis and correlating with cancer aggressiveness through enhancement of tumor cell proliferation and metastasis with poor clinical outcomes [38,41,42,43,46].

Although nicotine’s signaling through its receptors is well-defined, how cAMP-dependent protein kinase (PKA) affects the levels of VEGF induced by nicotine treatment and how this might be modulated by HA–CD44 and p53 status, remains unclear in NSCLC. While prior studies highlighted nicotine-induced survival signaling differences between A549 (p53^+^/^+^) and H1299 (p53-null) cells, whether PKA links receptor activation to secreted VEGF levels is not clear. HA synthesis inhibition, for example, with 4-methylumbelliferone (4-MU), can dampen oncogenic signaling in several cancers [49], suggesting a potentially unexplored crosstalk with the nicotine–PKA–VEGF axis in lung cancer. 

In this study, we tested the hypothesis that nicotine, acting through α7 nAChRs and/or β-ARs, activates PKA to drive increased VEGF levels in the conditioned media, in a manner regulated by HA–CD44 signaling and p53. We tested this hypothesis by performing the following: (i) manipulating PKA activity (PKI 14–22 amide; 8-Br-cAMP), (ii) blocking HA synthesis (4-MU) to modulate HA–CD44, and (iii) modulating p53 (inhibition/activation), then quantifying VEGF protein in the conditioned media. Functional outcomes were subsequently evaluated through immunodepletion of VEGF, followed by rescue experiments using the addition of purified VEGF protein. This design links nicotine-induced receptor activation to PKA signaling, engagement of the HA–CD44 pathway, and constraint by p53, culminating in elevated secreted VEGF and, consequently, greater cell survival with reduced apoptosis in NSCLC cells.

## 2. Results and Discussion

### 2.1. PKA Regulates the Levels of VEGF in the Media of A549 and H1299 Cells Untreated or Treated with Nicotine

Previously, we reported that VEGF signaling is regulated by nicotine in a manner dependent on nAChRs and/or β-ARs in NSCLC cells [21]. In this study, we set out to examine potential molecular players involved in the regulation of VEGF by nicotine in NSCLC cells. In lung cancer cells, the signaling pathways downstream of nAChRs are known to promote proliferation, in part, by mediating Ca^2+^-dependent activation of PKA and the induction of transcription factors that include CREB [6,7,8,15]. Classical cyclic AMP-dependent PKA activation is widely recognized to enhance cell survival signaling by upregulating the transcription of several oncogenes [50,51,52,53]. Adenylate cyclase was shown to activate the formation of cyclic AMP and PKA, leading to increased VEGF levels, stimulating growth, metastasis, and drug resistance of different types of cancer [30,31,54,55]. Treating osteoblast-like MC3T3-E1 cells with the cAMP analog 8-Br-cAMP, an activator of PKA, was reported to significantly increase the production and secretion of VEGF, promoting angiogenesis [56]. VEGF protein production was also increased by treatment of human airway smooth muscle cells with 8-Br-cAMP in a concentration-dependent manner [57]. 

**Figure 1:**
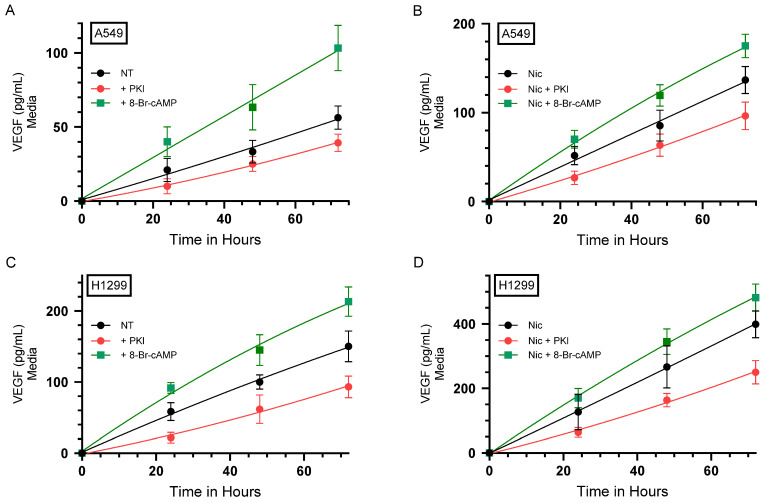

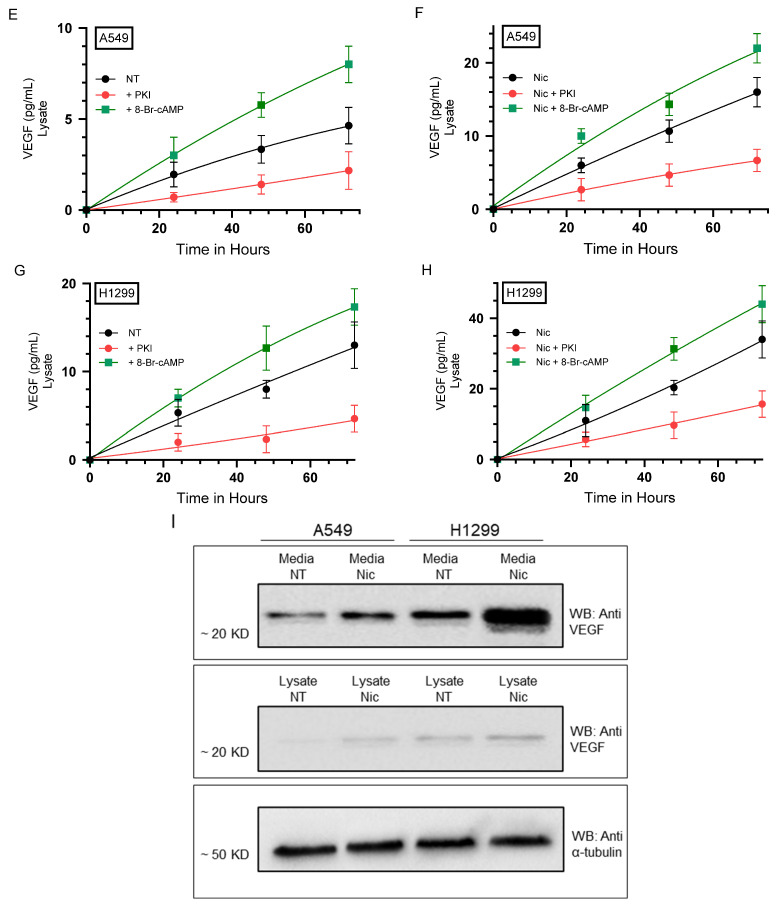
The levels of VEGF in the media of A549 and H1299 cells, untreated or treated with nicotine, are regulated by PKA. Cells (0.2 × 10^5^) were grown in media supplemented with 10% FBS overnight. The cell monolayers were then incubated in serum-free media for 24 h, then the media was replaced with fresh serum-free media (0 h). The cells were then either not treated (NT) (**A**,**C**,**E**,**G**) or treated with Nic (1 µM) (**B**,**D**,**F**,**H**), PKI 14-22 amide (5 µM), 8-Br-cAMP (500 µM), or in combination. The media and lysates were used to quantitate the levels of VEGF (Methods, *n* = 5), using the same concentration of protein (3 µL of 600 µg/mL total protein), as a function of time. Data were plotted using the GraphPad 10.6.0 software. Data shown as mean ± SD; *n* = 5 biological replicates per time point. Technical replicates were averaged within each biological replicate before analysis. Significance was set at *p* < 0.05; results with *p* < 0.01 were considered highly significant. Exact *p*-values are reported in Table 1; statistical procedures are detailed in Methods (Statistical analysis). Western blotting (**I**) using the indicated antibodies was carried out on the same concentration of total protein (80 µL of 600 µg/mL).

To test whether PKA is involved in the nicotine mediated increase in VEGF levels in NSCLC, cells were grown in media supplemented with serum overnight. The cell monolayers were then incubated in serum-free media for 24 h, then the media was replaced with fresh serum-free media. The cells were then either not treated or treated with nicotine, the PKA inhibitor (PKI 14-22 amide), the PKA activator (8-Br-cAMP), or in combination (Figure 1, Table 1). The levels of VEGF in the media of A549 cells increased over 72 h (Figure 1A). A549 cells treated with 8-Br-cAMP for 72 h had an ~1.85-fold increase in the levels of VEGF, while cell treatment with PKI decreased those levels ~1.45-fold relative to control untreated cells (Figure 1A). Consistent with our previous finding [21], A549 cell treatment with nicotine for 72 h increased the levels of VEGF in the media ~2.45-fold relative to control untreated cells (Figure 1A,B). These levels increased ~1.25-fold upon A549 cell treatment for 72 h with 8-Br-cAMP and decreased ~1.45-fold upon treatment with PKI (Figure 1B) relative to cells treated with only nicotine. 

The levels of VEGF in the media of H1299 cells also increased over 72 h (Figure 1C). H1299 cells treated with 8-Br-cAMP resulted in the ~1.40-fold increase in VEGF levels at 72 h compared to untreated controls, while cell treatment with PKI decreased those levels ~1.60-fold (Figure 1C). H1299 cells treated for 72 h with nicotine increased the levels of VEGF in the media ~2.65-fold relative to control untreated cells (Figure 1C,D), as we found earlier [21]. These levels increased ~1.20-fold upon H1299 cell treatment with 8-Br-cAMP and decreased ~1.60-fold upon treatment with PKI relative to nicotine treated cells (Figure 1D). These results show that PKA is an important regulator of the levels of VEGF in A549 and H1299 cell media. 

Prior NSCLC studies have already established intracellular and gene-level regulation of VEGF after nicotine treatment [58,59,60]. For example, in A549 cells, nicotine increased HIF-1α and VEGF, with VEGFA measured at the mRNA and protein levels, and conditioned media from nicotine-treated A549 cells was shown to drive functional angiogenesis (human umbilical vein endothelial cells (HUVEC) tube formation), effects that diminished with HIF-1α blockade or Epigallocatechin-3-gallate treatment [58,59]. In xenografts, exposure to nicotine (with estradiol) was reported to elevate tumor VEGF and microvessel density, linking transcriptional induction to angiogenic output in vivo [60]. Side-by-side studies in A549 and H1299 cells further show that nicotine raises VEGF levels in the conditioned media and that this response is suppressed by α-BTX, propranolol, and/or dihydro-β-erythroidine, indicating nicotinic/β-adrenergic control upstream of VEGF in NSCLC [21]. Complementary work with cigarette-smoke extract (a nicotine-containing stimulus) demonstrated HIF-1α–dependent VEGFA mRNA induction in A549 cells, reinforcing the transcriptional mechanism [61]. Consistent patterns are seen in other cancers. For example, in gastric cancer, nicotine induces COX-2 and VEGF and increases angiogenesis in sponge/Matrigel assays (attenuated by COX-2 or VEGFR inhibition); in nasopharyngeal carcinoma, nicotine shifts the VEGF/PEDF balance via α7-nAChR/ERK/HIF-1α with VEGF measured by PCR and protein assays; and in esophageal cancer, nicotine up-regulates VEGF-C mRNA and protein via an OTUD3/ZFP36 axis that drives lymphatic metastasis [20,62,63]. Together, these reports document protein and mRNA regulation of VEGF and its functional consequences after nicotine exposure in NSCLC and other tumor systems.

VEGF is a secreted protein, traditionally purified and quantitated in tumor-cell conditioned media [64]. In NSCLC models, nicotine increases HIF-1α and VEGF in A549 and H157 cells, with VEGF routinely quantified in the conditioned media by ELISA and linked to functional angiogenesis (e.g., HUVEC tube formation) [59]. Experiments further show that nicotine elevates VEGF in the conditioned media from A549 and H1299 cells, and that this response is blocked by α-BTX, propranolol, and dihydro-β-erythroidine, indicating nicotinic/β-adrenergic control of the secreted VEGF pool in both cell lines [21]. Since VEGF is rapidly secreted, its intracellular levels are typically difficult to measure by ELISA unless secretion is blocked pharmacologically (e.g., with Brefeldin A), which allows intracellular accumulation to be measured more easily [65]. Parallel observations in other cancers support this framework. For example, in nasopharyngeal carcinoma, nicotine increases VEGF expression (mRNA and protein levels) and shifts the VEGF/PEDF balance, while secreted VEGF is quantified from the conditioned media [20]. 

The levels of VEGF in A549 cell lysates in the absence of nicotine treatment represented ~12% of the levels found in the media after 72 h of incubation (Figure 1A,E). The levels of VEGF in A549 cell lysates upon nicotine treatment represented ~8.5% of the levels found in the media after 72 h of incubation (Figure 1B,F). Similarly, the levels of VEGF in H1299 cell lysates in the absence of nicotine treatment represented ~13% of the levels found in the media after 72 h of incubation (Figure 1C,G). The levels of VEGF in H1299 cell lysates upon nicotine treatment represented ~11.7% of the levels found in the media after 72 h of incubation (Figure 1D,H). In both cell lines, the effects of the PKA inhibitor and activator on the levels of VEGF in the cell lysates mirrored those observed in the media (Figure 1A–H), and Western blotting corroborated the relative abundances (Figure 1I). Together, these data indicate that, under our assay conditions, VEGF is predominantly found in the conditioned media, with intracellular levels constituting only a small minority of total VEGF. Accordingly, and to be consistent with prior studies that center quantitation on the secreted pool, the experiments in this study focus on quantitating VEGF concentrations in the conditioned media since the concentration of intracellular VEGF was found to be significantly lower relative to that found in the conditioned media (Figure 1). 

### 2.2. The Levels of VEGF Decreased upon Blocking PKA Activity and HA Synthesis in Both Cell Lines and Increased upon Blocking p53 Activity in A549 Cells

A significant reduction in the levels of VEGF was observed in the serum from mice treated with 4-Methylumbelliferone (4-MU) [66]. Treatment with 4-MU, known to inhibit HA synthases, decreased the accumulation of HA in the ECM, blocked CD44 activation, and resulted in antimetastatic and proapoptotic effects in cultured tumor cells [49,67,68,69]. A positive relationship was shown between the expression of CD44 and VEGF in head and neck carcinomas [70]. Activation of HA-CD44 signaling was also reported to increase VEGF expression in endothelial cells and cancer cells, activating the VEGF pathway and increasing endothelial cell proliferation [71]. Previously, we reported that nicotine treatment of NSCLC cells upregulated the levels of VEGF, while p53 led to downregulation of VEGF levels [21]. 

**Figure 2:**
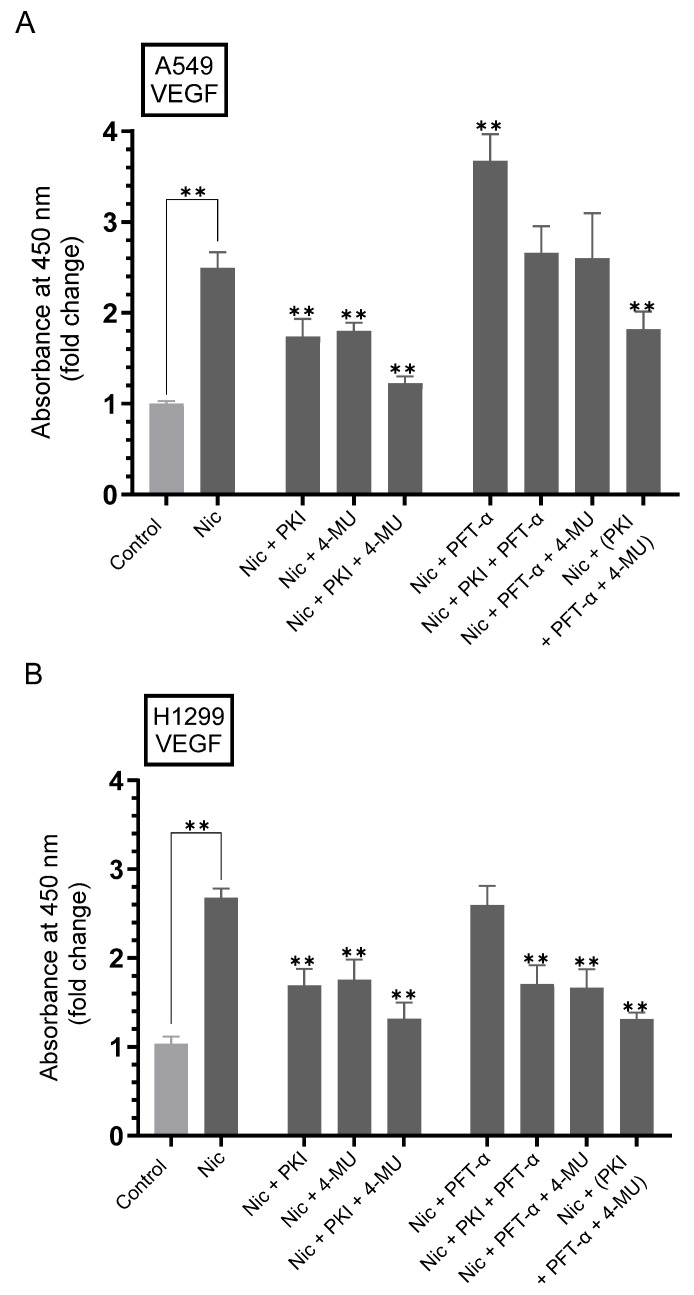
Blocking PKA activity and HA synthesis decreased the levels of VEGF in both cell lines, while the opposite was observed upon inhibiting the p53 activity in A549 cells. Cells were grown in FBS-supplemented media for 24 h, serum starved overnight, then incubated for 72h with +/− Nic (1 µM), PKI 14-22 amide (5 µM), 4-MU (600 µM), PFT-α (10 μM), or in combination. The levels of VEGF in the media of A549 cells (**A**) and H1299 cells (**B**) were measured as described in the Methods section. The graphs summarize the results expressed as means ± S.D. of three separate experiments, each performed in triplicate. Each condition reflects *n* = 3 biological replicates per time point. Technical replicates were averaged within each biological replicate before analysis. Asterisks indicate a statistically significant difference relative to nicotine-treated cells, Mann–Whitney test, ** *p* < 0.0l. Absence of asterisks indicates no significance.

Based on these reports, we examined the effects of blocking PKA, HA levels and signaling, p53, alone or in combination, on the levels of VEGF in the media of nicotine-treated A549 and H1299 cells (Figure 2, Table 2). Cells were grown in FBS-supplemented media overnight, serum starved for 24 h, then incubated for 72 h with +/− nicotine, PKI (PKA inhibitor), 4-MU, pifithrin-α (PFT-α, an inhibitor of p53). Treatment of A549 cells with nicotine and either PKI or 4-MU decreased the levels of VEGF ~1.40-fold compared to cells treated with only nicotine, while co-incubation with both PKI and 4-MU resulted in a ~2.00-fold decrease in VEGF levels (Figure 2A). Treatment of A549 cells with nicotine and PFT-α led to an ~1.50-fold increase in VEGF levels (Figure 2A) compared to nicotine-treated A549 cells. Compared to A549 cells treated with nicotine + PFT-α, the levels of VEGF decreased by co-incubation with nicotine + PKI + PFT-α and nicotine + PFT-α + 4-MU (~1.40-fold), nicotine + PKI + PFT-α + 4-MU (~2.00-fold) (Figure 2A). Treatment of H1299 cells with nicotine and either PKI or 4-MU decreased the levels of VEGF ~1.55-fold compared to cells treated with only nicotine, while co-incubation with both PKI and 4-MU resulted in a ~2.00-fold decrease in VEGF levels (Figure 2B). No difference in the levels of VEGF was found upon treatment of H1299 cells with PFT-α compared to any of the cell treatments in the absence of PFT-α, a result not surprising since H1299 cells are known to be p53-null (Figure 2B). 

### 2.3. Nicotine-Induced PKA Activation Decreased by Co-Treatment with α-Btx and/or Prop, While HA Levels Correlated with the Activity of PKA

The β2-AR was found to be involved in NSCLC progression [25,33,72,73], and using HEK293 cells, HA production was shown to increase by β2-AR activation due to increased HA synthase 2 (HAS2) expression via the PKA signaling pathway [74].

**Figure 3:**
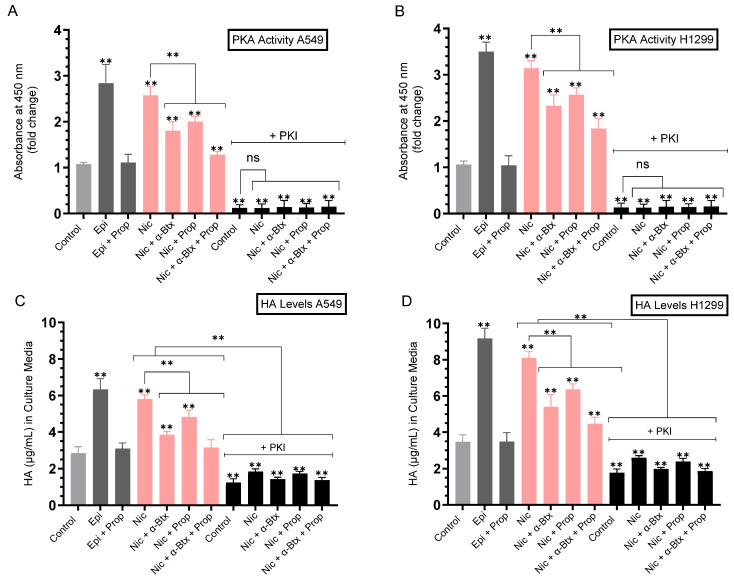
PKA activity in cells increased upon treatment with nicotine, an effect decreased by co-treatment with α-Btx and/or Prop, while HA levels increased in parallel to PKA activation and decreased upon PKA inhibition. Cells were grown in FBS-supplemented media for 24 h, serum starved overnight, then incubated for 72h with +/− Epi (100 nM), Prop (1 µM), Nic (1 µM), α-Btx (1 µM), PKI 14-22 amide (5 µM), or a combination. The PKA activity (**A**,**B**) and the levels of HA (**C**,**D**) were measured as described in the Methods section. The graphs summarize the results expressed as means ± S.D. of five separate experiments, each performed in triplicate. Each condition reflects *n* = 5 biological replicates per time point. Technical replicates were averaged within each biological replicate before analysis. Asterisks indicate a statistically significant difference relative to untreated control without PKI, Mann–Whitney test. Statistical differences between different groups were analyzed by ANOVA followed by Tukey’s post hoc multiple comparison test, ** *p* < 0.0l. Absence of asterisks indicates no significance (ns).

To examine the effect of nicotine and the involvement of the α7nAChRs and β-AR on the activity of PKA and HA levels, and whether blocking the activity of PKA affects the levels of HA, cells were grown in FBS-supplemented media for 24 h, serum starved overnight, then either untreated or incubated for 72 h with epinephrine (the non-selective agonist of all adrenergic receptors), propranolol (the non-selective β-blocker), nicotine, α-Btx (the competitive antagonist that binds with high affinity to α7nAChRs), PKI 14-22 amide (PKA inhibitor), or in combination (Figure 3, Table 3).

Treatment of A549 cells with epinephrine increased the PKA activity ~2.85-fold, while co-treatment with both epinephrine and propranolol blocked this effect (Figure 3A). Relative to the nicotine-induced increase in the PKA activity in A549 cells, ~2.55-fold, co-treatment with nicotine and α-Btx decreased the activity of PKA ~1.40-fold, co-treatment with nicotine and propranolol decreased the activity of PKA ~1.25-fold, while co-treatment with nicotine, α-Btx, and propranolol resulted in a ~2.00-fold decrease in the activity of PKA (Figure 3A).

Similar trends were found in H1299 cells (Figure 3B). Treatment of H1299 cells with epinephrine increased the PKA activity ~3.50-fold, an effect blocked by co-treatment with both epinephrine and propranolol (Figure 3B). Relative to the nicotine-induced increase in the PKA activity in H1299 cells, ~3.15-fold, co-treatment with nicotine and α-Btx decreased the activity of PKA ~1.35-fold, co-treatment with nicotine and propranolol decreased the activity of PKA ~1.20-fold, while co-treatment with nicotine, α-Btx, and propranolol resulted in a ~1.70-fold decrease in the activity of PKA (Figure 3B). As expected, the PKA activity was effectively blocked in both cell lines by the PKA inhibitor, PKI (Figure 3A,B).

The finding that co-treatment of A549 and H1299 cells with nicotine + α-Btx + propranolol is more effective at decreasing PKA activity compared to nicotine with either α-Btx or propranolol suggests that a mechanism by which nicotine activates PKA involves signaling through α7nAChRs, and β-AR (Figure 3A,B).

As observed for the PKA activity, similar trends were found for the levels of HA, measured using the same treatments according to methods we used previously [75] (Figure 3C,D).

Treatment of A549 cells with epinephrine increased the levels of HA ~2.20-fold, while co-treatment with both epinephrine and propranolol blocked this effect (Figure 3C), suggesting the involvement of the β-ARs in regulating HA levels. Relative to the nicotine-induced increase in the levels of HA in A549 cells compared to control, ~2.00-fold, co-treatment with nicotine and α-Btx decreased the HA levels ~1.50-fold, co-treatment with nicotine and propranolol decreased the levels of HA ~1.20-fold, while co-treatment with nicotine, α-Btx, and propranolol resulted in a ~1.85-fold decrease in the levels of HA (Figure 3C). Blocking the PKA activity using PKI in the absence of nicotine decreased the levels of HA ~2.30-fold compared to control and reduced the effects of nicotine (~1.50-fold increase compared to control cells treated with PKI), suggesting the likely involvement of PKA in nicotine-independent and -dependent regulation of HA levels (Figure 3C). In the presence of PKI, relative to A549 cells treated with nicotine, co-treatment with nicotine and α-Btx decreased the HA levels ~1.28-fold, co-treatment with nicotine and propranolol decreased the levels of HA ~1.05-fold, while co-treatment with nicotine, α-Btx, and propranolol resulted in a ~1.34-fold decrease in the levels of HA (Figure 3C).

Treatment of H1299 cells with epinephrine increased the levels of HA ~2.65-fold, while co-treatment with both epinephrine and propranolol abolished this effect (Figure 3D). Relative to the nicotine-induced increase in the levels of HA in H1299 cells compared to control, ~2.30-fold, co-treatment with nicotine and α-Btx decreased the HA levels ~1.48-fold, co-treatment with nicotine and propranolol decreased the levels of HA ~1.25-fold, while co-treatment with nicotine, α-Btx, and propranolol resulted in a ~1.75-fold decrease in the levels of HA (Figure 3D). Blocking the PKA activity using PKI in the absence of nicotine decreased the levels of HA ~1.95-fold compared to control and reduced the effects of nicotine (~1.45-fold increase compared to control cells treated with PKI), suggesting the likely involvement of PKA in nicotine-independent and -dependent regulation of HA levels (Figure 3D). In the presence of PKI, relative to H1299 cells treated with nicotine, co-treatment with nicotine and α-Btx decreased the HA levels ~1.30-fold, co-treatment with nicotine and propranolol decreased the levels of HA ~1.08-fold, while co-treatment with nicotine, α-Btx, and propranolol resulted in a ~1.40-fold decrease in the levels of HA (Figure 3D).

### 2.4. Opposite Effects Were Observed on the Activity of p53 in A549 Cells and the Levels of VEGF in the Media upon Treatment with Nicotine, an Effect Decreased by Co-Treatment with α-Btx and/or Prop, and upon PKA Inhibition

Epinephrine was reported to act through the β2-AR to activate the PKA pathway, promoting AKT-mediated activation of the negative regulator of p53, MDM2 [76]. Increased levels of the PKA-catalytic subunit were observed in p53-deficient cells [77], while activation of PKA inhibited the activity of p53 [78]. Lung cancer cells were found to express β-ARs that function to facilitate apoptosis resistance and cellular proliferation [25]. β-ARs are well established to induce the release of VEGF [50], which can contribute to the development, progression, and resistance to cancer targeted therapies. Previously, we reported that VEGF can be upregulated via α7nAChR and/or β-ARs and downregulated via p53 and/or GABA in response to NSCLC cell treatment with nicotine [21]. 

In A549 cells, nicotine acting through the α7nAChRs was reported to activate PI3K–AKT/ERK signaling, which is known to enhance MDM2 activity and thereby suppress p53 stability and transcriptional output [6,79,80]. Mechanistically, AKT- and ERK-driven phosphorylation of MDM2 promotes p53 degradation and inactivation, providing a clear route by which nicotine can decrease p53 activity in NSCLC [80,81]. Studies directly comparing A549 and H1299 cells show that nicotine-driven survival signaling is more pronounced when the function of p53 is impaired or absent, and that restoring or silencing p53 correspondingly shifts nicotine’s effects [82]. Similar effects of the pathway from nAChR through AKT and ERK to MDM2 and p53 have been reported across other cancers (head and neck, pancreatic, breast, and colorectal), where nicotine or nAChR stimulation activates pro-survival signaling and functionally suppresses p53 [5,83,84]. 

**Figure 4:**
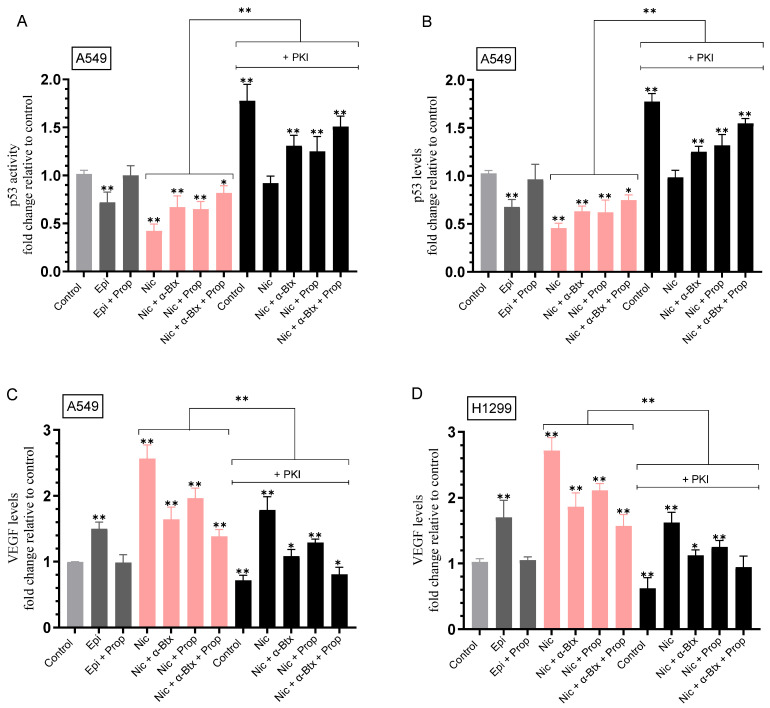
Treatment with nicotine resulted in opposite effects on the activity of p53 in A549 cells and the levels of VEGF in the media, an effect decreased by co-treatment with α-Btx and/or Prop, and upon PKA inhibition. Cells were grown in FBS-supplemented media for 24h, serum starved overnight, then incubated for 72h with +/− Epi (100 nM), Prop (1 µM), Nic (1 µM), α-Btx (1 µM), PKI 14-22 amide (5 µM), or a combination. The activity of p53 in A549 cell lysates (**A**) and p53 levels (**B**) and the levels of VEGF in the media of A549 (**C**) and H1299 (**D**) cells were measured as described in Methods. The graphs summarize the results expressed as means ± S.D. of five separate experiments, each performed in triplicate. Each condition reflects *n* = 5 biological replicates per time point. Technical replicates were averaged within each biological replicate before analysis. Asterisks indicate a statistically significant difference relative to untreated control without PKI, Mann–Whitney test. Statistical differences between different groups were analyzed by ANOVA followed by Tukey’s post hoc multiple comparison test, * *p* < 0.05, ** *p* < 0.01. Absence of asterisks indicates no significance (ns).

To examine the effect of PKA on the activity of p53 in A549 cells and the levels of VEGF in the media, A549 and H1299 cells were grown in FBS-supplemented media overnight, serum starved for 24 h, then incubated for 72 h in the absence or presence of epinephrine, propranolol, nicotine, α-Btx, PKI 14-22 amide, or in combination (Figure 4, Table 4). The activity of p53 in A549 cell lysates and the levels of VEGF in the media of A549 and H1299 cells were measured as described in Methods. 

Treatment of A549 cells with epinephrine decreased the p53 activity ~1.40-fold, an effect abolished upon co-treatment with propranolol (Figure 4A). Relative to A549 control, nicotine decreased the p53 activity ~2.40-fold (Figure 4A). Relative to A549 cells treated with nicotine, co-treatment with nicotine and α-Btx or propranolol increased the p53 activity ~1.60-fold, while co-treatment with nicotine, α-Btx, and propranolol resulted in a ~1.95-fold increase in the activity of p53 (Figure 4A). Blocking the PKA activity using PKI in the absence of nicotine increased the p53 activity ~1.75-fold compared to control. Relative to A549 cells treated with nicotine, co-treatment with nicotine and PKI increased the p53 activity ~2.20-fold. Relative to A549 cells co-treated with nicotine and PKI, the activity of p53 increased ~1.40-fold upon co-treatment with nicotine and α-Btx, 1.35-fold with co-treatment with nicotine and propranolol, and ~1.65-fold upon co-treatment with nicotine, α-Btx, and propranolol (Figure 4A). Almost identical results and trends were found when the levels of p53 were measured under the same conditions (Figure 4B).

Opposite trends were observed on the levels of VEGF under the same conditions (Figure 4C). Treatment of A549 cells with epinephrine increased the levels of VEGF ~1.50-fold, an effect abolished by co-treatment with propranolol (Figure 4C). Relative to A549 control, nicotine increased the VEGF levels ~2.55-fold (Figure 4C). Relative to A549 cells treated with nicotine, co-treatment with nicotine and α-Btx reduced the VEGF levels ~1.55-fold, co-treatment with nicotine and propranolol led to a ~1.30-fold decrease in the levels of VEGF, while co-treatment with nicotine, α-Btx, and propranolol resulted in a ~1.85-fold decrease in VEGF levels (Figure 4C). Blocking the PKA activity using PKI in the absence of nicotine decreased the levels of VEGF ~1.40-fold compared to control. Relative to A549 cells treated with nicotine, co-treatment with nicotine and PKI decreased the VEGF levels ~1.45-fold (Figure 4C). Relative to A549 cells co-treated with nicotine and PKI, VEGF levels decreased ~1.65-fold upon co-treatment with nicotine and α-Btx, 1.35-fold with co-treatment with nicotine and propranolol, and ~2.20-fold upon co-treatment with nicotine, α-Btx, and propranolol (Figure 4C).

Treatment of H1299 cells with epinephrine increased the levels of VEGF ~1.70-fold, an effect blocked by co-treatment with propranolol (Figure 4D). Relative to H1299 control, nicotine increased the VEGF levels ~2.70-fold (Figure 4D). Relative to H1299 cells treated with nicotine, co-treatment with nicotine and α-Btx reduced the VEGF levels ~1.45-fold, co-treatment with nicotine and propranolol led to a ~1.30-fold decrease in the levels of VEGF, while co-treatment with nicotine, α-Btx, and propranolol resulted in a ~1.70-fold decrease in VEGF levels (Figure 4D). Blocking the PKA activity using PKI in the absence of nicotine decreased the levels of VEGF ~1.60-fold compared to control. Relative to H1299 cells treated with nicotine, co-treatment with nicotine and PKI decreased the VEGF levels ~1.70-fold (Figure 4D). Relative to H1299 cells co-treated with nicotine and PKI, VEGF levels decreased ~1.40-fold upon co-treatment with nicotine and α-Btx, 1.30-fold with co-treatment with nicotine and propranolol, and ~1.70-fold upon co-treatment with nicotine, α-Btx, and propranolol (Figure 4D). Collectively, these results show that blocking the PKA activity has opposite effects on the p53 activity and levels of VEGF in A549 cells and suggest the likely involvement of PKA in nicotine-independent and -dependent regulation of VEGF levels in both cell lines.

### 2.5. Knockdown of p53 Increased the PKA Activity and the Levels of VEGF in the Media of A549 Cells Untreated or Treated with Nicotine

**Figure 5:**
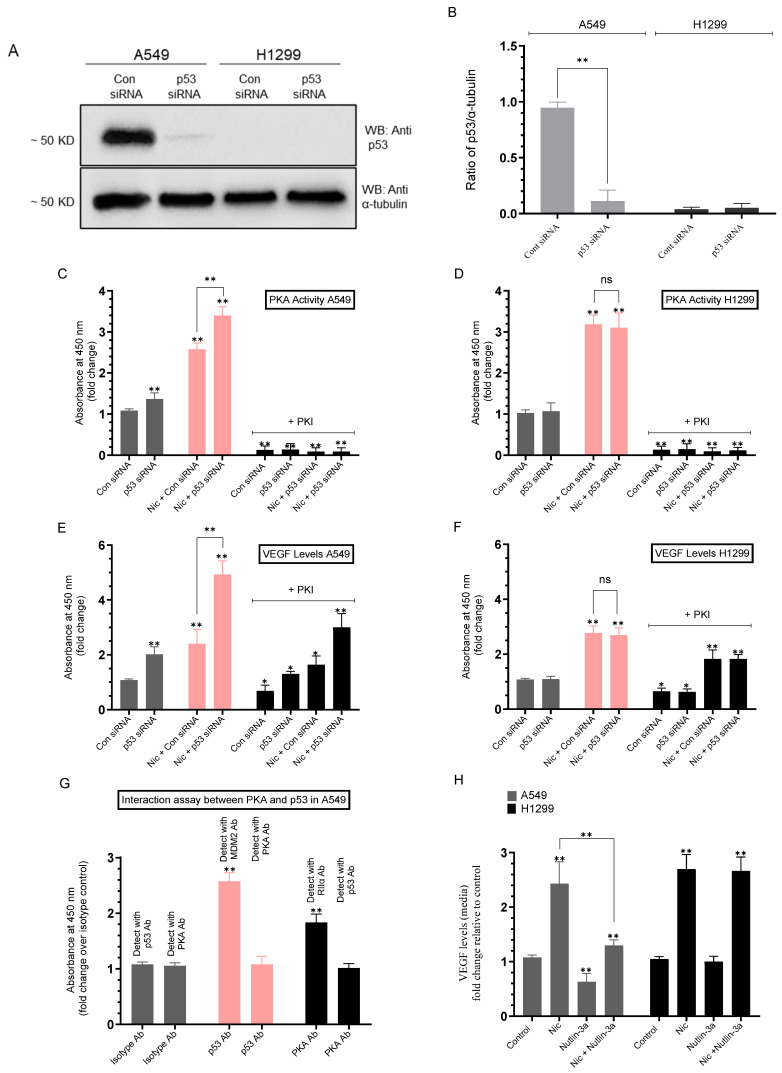
The PKA activity and the levels of VEGF in the media increased in A549 cells untreated or treated with nicotine upon p53 knockdown. Cells (0.2 × 10^5^) were grown in media supplemented with 10% FBS overnight. The cell monolayers were then incubated in serum-free media for 24 h, then the media was replaced with fresh serum-free media. The cells were then incubated with the indicated siRNAs and either not treated or treated with nicotine (Nic, 1 µM) for 72 h (Methods). Western blotting (**A**) and quantitation (**B**) using the indicated antibodies were carried out on the same concentration of total protein (80 µL of 600 µg/mL) of the cell lysates. The PKA activity in A549 (**C**) and H1299 (**D**) cells was measured (Methods). The media was used to quantitate the levels of VEGF in A549 cells (**E**) and H1299 cells (**F**) as described in the Methods section (*n* = 3), using the same concentration of protein (3 µL of 600 µg/mL total protein). (**G**) shows an ELISA-based interaction assay where the indicated antibodies are immobilized first to the wells (X-axis), followed by detection using the antibodies shown on top of the bar graphs. (**H**) shows the levels of VEGF in the media in the absence or presence of nicotine (Nic, 1 µM), Nutlin-3a (10 μM), or in combination. Each condition reflects *n* = 3 biological replicates per time point. Technical replicates were averaged within each biological replicate before analysis. Data were plotted using the GraphPad 10.6.0 software. ** *p* < 0.01. Absence of asterisks indicates no significance (ns).

We next tested whether knockdown of p53 affects the activity of PKA and the levels of VEGF (Figure 5, Table 5). Cells were grown in media supplemented with serum overnight. The cell monolayers were then incubated in serum-free media for 24 h, then the media was replaced with fresh serum-free media. The cells were then incubated with the indicated siRNAs (Figure 5) and either not treated or treated with nicotine. The PKA activity and the levels of VEGF were then measured as described in the Methods section.

Treatment of A549 cells with p53 siRNA (Figure 5A,B) led to a ~1.35-fold increase in the PKA activity in untreated cells compared to control and a ~1.30-fold increase in nicotine treated cells (Figure 5C). No effects were observed in H1299 cells, which is expected since they are known to be p53-null (Figure 5D). Previously, we reported that the p53 activity was decreased by treatment of A549 cells with nicotine, an effect that was partially reversed by using media immunodepleted of VEGF [21]. Treatment of A549 cells with p53 siRNA led to an ~2.00-fold increase in the levels of VEGF in untreated or nicotine-treated cells compared to cells transfected with control siRNA (Figure 5E), a finding consistent with our previous report [21]. Treatment of A549 cells with control siRNA and PKI decreased the levels of VEGF ~1.45-fold compared to control in the absence of PKI. Treatment of A549 cells with p53 siRNA and PKI decreased the levels of VEGF ~1.55-fold compared to cells transfected with p53 siRNA in the absence of PKI. Treatment of A549 cells with control siRNA, nicotine, and PKI decreased the levels of VEGF ~1.45-fold compared to control in the absence of PKI. Treatment of A549 cells with p53 siRNA, nicotine, and PKI decreased the levels of VEGF ~1.65-fold compared to cells transfected with p53 siRNA and treated with nicotine in the absence of PKI (Figure 5E).

Treatment of H1299 cells with nicotine increased the levels of VEGF ~2.70-fold in the media of cells transfected with either control or p53 siRNA (Figure 5F). Treatment of H1299 cells with control or p53 siRNA and PKI decreased the levels of VEGF ~1.55-fold compared to control in the absence of PKI. Treatment of H1299 cells with control or p53 siRNA, nicotine, and PKI decreased the levels of VEGF ~1.60-fold compared to the same treatment in the absence of PKI (Figure 5E). While these results show that p53 acts to inhibit PKA activity, the regulation of the levels of VEGF can occur independently of p53.

Our results show that nicotine signaling elevates pro-survival pathways and reduces p53 activity in A549 cells (Figure 4). Since H1299 cells are known to be p53-null, activation of pro-survival pathways by nicotine appears to also be p53-independent. Mechanistically, many kinases regulate p53 by phosphorylation, altering its stability and transcriptional output [83,84]. With respect to PKA, classic biochemical work demonstrated that PKA can phosphorylate p53 in vitro in a conformation- and concentration-dependent manner, as an enzyme–substrate encounter rather than a canonical, stable binding partner [85]. Thus, while PKA signaling could modulate p53 function, a strong, constitutive PKA–p53 complex is not considered part of the core p53 interactome as that found for MDM2–p53. In cancer more broadly, nAChR/cAMP/PKA pathways intersect with other networks such as MAPK/AKT signaling that can influence p53 indirectly [19]. In A549 cells, cAMP–PKA signaling intersects with the p53 pathway largely in a way that dampens stress-induced p53 activation, and elevating cAMP in lung cancer models was reported to increase p53–MDM2 binding, thereby reducing p53 activity [86]. 

To test whether PKA and p53 are found in a complex under our conditions in A549 cells, we used an ELISA-based interaction assay where p53 antibodies (mouse) were coated in the wells followed by detection using PKA antibodies (rabbit) and, conversely, coating wells with PKA antibodies and then detecting the presence of p53 using anti-p53 antibodies (Figure 5G). MDM2 and RIIα were used as positive controls for p53 and PKA, respectively, while isotype IgG antibodies were used as a negative control. No interaction was found between PKA and p53 (Figure 5G). These results suggest that PKA does not form a complex with p53 in A549 cells but is likely to operate through other transient encounters and pathway crosstalk rather than via binding interaction in A549 cell lysates. 

Consistent with a p53 brake on pro-angiogenic signaling, nutlin-3a (an MDM2 antagonist that stabilizes p53) has been shown to reduce HIF-1α function, dampening VEGF expression in a manner involving p53, and exerting anti-angiogenic effects more broadly [87,88]. To test whether the levels of VEGF in the media are also affected by activation of p53 in A549 cells, cells were either untreated or treated with nicotine, nutlin-3a, or a combination (Figure 5H). Relative to control A549 cells, treatment with nicotine increased the levels of VEGF in the media ~2.40-fold. Treatment of A549 cells with nutlin-3a decreased the levels of VEGF ~1.60-fold compared to control, while co-treatment with nicotine and nutlin-3a decreased VEGF levels ~1.85-fold compared to nicotine-treated cells (Figure 5H). No effects were observed when using nutlin-3a in H1299 cells, which is expected since H1299 cells are p53-null (Figure 5H). These results show that activating p53 with nutlin-3a counteracts nicotine-driven VEGF increases in A549 cells. 

### 2.6. Co-Treatment with Nicotine and Either PKI, 4-MU, VEGF Antibodies, or in Combination Resulted in Decreased Cell Viability and Increased Apoptosis Compared to A549 and H1299 Cell Treatment Using Only Nicotine

**Figure 6:**
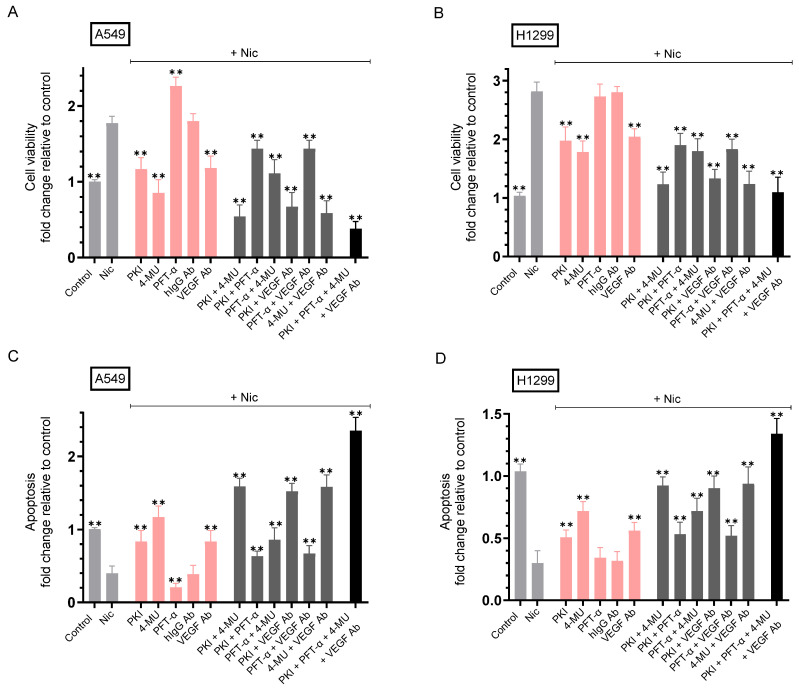
Compared to A549 and H1299 cell treatment with only nicotine (Nic), co-treatment with Nic and either PKI, 4-MU, VEGF Ab, or a combination resulted in decreased cell viability and increased apoptosis. Cells were grown in media with 10% FBS for 24 h, serum-starved overnight, then incubated in serum-free media for 72h in the absence or presence of Nic (1 µM), PKI 14-22 amide (5 µM), 4-MU (600 µM), PFT-α (10 μM), hIgG (20 μg/mL) as a control, anti-VEGF-specific antibodies (20 μg/mL), or a combination. Cell viability (**A**,**B**) and apoptosis (**C**,**D**) were measured as described in the Methods section. Data were averaged, normalized, and expressed as a fold change relative to cells treated with only Nic of each cell line using the GraphPad 10.6.0 software. The graphs summarize the results expressed as means ± S.D. of three separate experiments, each performed in triplicate. Each condition reflects *n* = 3 biological replicates per time point. Technical replicates were averaged within each biological replicate before analysis. Asterisks indicate a statistically significant difference relative to untreated control, Mann–Whitney test, ** *p* < 0.01. Absence of asterisks indicates no significance.

To test the effect of the inhibitors on cell viability and apoptosis, cells were grown in media with 10% FBS for 24 h, serum-starved overnight, then incubated in serum-free media for 72 h in the absence or presence of nicotine, the inhibitors (PKI 14-22 amide, 4-MU, PFT-α), anti-VEGF-specific antibodies, or in combination (Figure 6, Table 6). 

Treatment of A549 cells with nicotine increased cell viability ~1.75-fold compared to control untreated cells (Figure 6A). In the presence of nicotine, A549 cell viability decreased ~1.53-fold by co-incubation with PKI and ~2.00-fold by co-incubation with 4-MU compared to cells treated with only nicotine (Figure 6A). Conversely, co-treatment of A549 cells with nicotine and PFT-α increased cell viability ~1.30-fold compared to nicotine treated cells, a finding in support of the tumor suppressor functions of p53. Neutralizing VEGF function using a VEGF neutralizing antibody previously used in NSCLC [89] that we also used [21], decreased A549 cell viability in the presence of nicotine ~1.53-fold compared to A549 cells treated with nicotine and hIgG control (Figure 6A). 

Co-treatment of A549 cells with nicotine + PKI + 4-MU decreased cell viability ~3.30-fold, an effect more pronounced compared to A549 cells treated with nicotine and either PKI or 4-MU (Figure 6A). Relative to nicotine treated cells, co-treatment of A549 cells with nicotine + PKI + PFT-α decreased cell viability ~1.23-fold, an effect less pronounced compared to A549 cells treated with nicotine and PFT-α but more pronounced compared to A549 cells treated with nicotine and PKI. A similar trend was observed upon co-treatment of A549 cells with nicotine + PFT-α + 4- MU which decreased cell viability ~1.60-fold relative to nicotine treated cells, an effect less pronounced compared to A549 cells treated with nicotine and PFT-α but more pronounced compared to A549 cells treated with nicotine and 4-MU (Figure 6A). Co-treatment of A549 cells with nicotine + PKI + VEGF antibody decreased cell viability ~2.65-fold, an effect more pronounced compared to A549 cells treated with nicotine and either PKI or the VEGF antibody. Co-treatment of A549 cells with nicotine + PFT-α and the VEGF antibody decreased cell viability ~1.23-fold relative to nicotine treated cells, an effect less pronounced compared to A549 cells treated with nicotine and PFT-α but more pronounced compared to A549 cells treated with nicotine and the VEGF antibody (Figure 6A). Co-treatment of A549 cells with nicotine + 4-MU + VEGF antibody decreased cell viability ~3.05-fold, an effect more pronounced compared to A549 cells treated with nicotine and either 4-MU or the VEGF antibody. The largest decrease in A549 cell viability, ~4.66-fold, was observed in cells treated with nicotine + PKI + PFT-α + 4-MU + the VEGF antibody (Figure 6A). 

Treatment of H1299 cells with nicotine increased cell viability ~2.80-fold compared to control untreated cells (Figure 6B). In the presence of nicotine, H1299 cell viability decreased ~1.43-fold by co-incubation with PKI and ~1.58-fold by co-incubation with 4-MU, while no effects were found upon addition of PFT-α or hIgG control, compared to cells treated with only nicotine. Addition of the neutralizing VEGF antibodies decreased H1299 cell viability in the presence of nicotine ~1.38-fold compared to H1299 cells treated with nicotine and hIgG control (Figure 6B). 

Co-treatment of H1299 cells with nicotine + PKI + 4-MU decreased cell viability ~2.28-fold compared to nicotine treated cells, an effect more pronounced compared to H1299 cells treated with nicotine and either PKI or 4-MU (Figure 6B). The effects observed by co-treatment of H1299 cells with nicotine + (PKI + PFT-α), (PFT-α + 4-MU), or (PFT-α + VEGF antibody) were indistinguishable from those in the absence of added PFT-α, consistent with the lack of p53 in this cell line (Figure 6B). Co-treatment of H1299 cells with nicotine + PKI + VEGF antibody decreased cell viability ~2.10-fold, an effect more pronounced compared to H1299 cells treated with nicotine and either PKI or the VEGF antibody. Co-treatment of H1299 cells with nicotine + 4-MU + VEGF antibody decreased cell viability ~2.26-fold, an effect more pronounced compared to H1299 cells treated with nicotine and either 4-MU or the VEGF antibody. H1299 cell viability decreased ~2.58-fold in cells treated with nicotine + PKI + PFT-α + 4-MU + the VEGF antibody (Figure 6B). 

Treatment of A549 cells with nicotine decreased apoptosis ~2.50-fold compared to control untreated cells (Figure 6C). In the presence of nicotine, A549 cell apoptosis increased ~2.10-fold by co-incubation with PKI and ~2.93-fold by co-incubation with 4-MU compared to cells treated with only nicotine (Figure 6C). Conversely, co-treatment of A549 cells with nicotine and PFT-α decreased apoptosis ~2.00-fold compared to nicotine treated cells. Co-treatment of A549 cells with nicotine and the neutralizing VEGF antibodies increased A549 cell apoptosis ~2.10-fold compared to A549 cells treated with nicotine and hIgG control (Figure 6C). 

Co-treatment of A549 cells with nicotine + PKI + 4-MU increased apoptosis ~4.00-fold compared to cells treated with only nicotine, an effect more pronounced compared to A549 cells treated with nicotine and either PKI or 4-MU (Figure 6C). Co-treatment of A549 cells with nicotine + PKI + PFT-α increased apoptosis ~1.58-fold relative to nicotine treated cells, an effect more pronounced compared to A549 cells treated with nicotine and PFT-α but less pronounced compared to A549 cells treated with nicotine and PKI. A similar trend was observed upon co-treatment of A549 cells with nicotine + PFT-α + 4-MU which increased apoptosis ~2.15-fold compared to cells treated with only nicotine, an effect more pronounced compared to A549 cells treated with nicotine and PFT-α but less pronounced compared to A549 cells treated with nicotine and 4-MU (Figure 6C). Co-treatment of A549 cells with nicotine + PKI + VEGF antibody increased apoptosis ~3.80-fold, an effect more pronounced compared to A549 cells treated with nicotine and either PKI or the VEGF antibody. Co-treatment of A549 cells with nicotine + PFT-α and the VEGF antibody increased apoptosis ~1.68-fold compared to cells treated with only nicotine, an effect more pronounced compared to A549 cells treated with nicotine and PFT-α but less pronounced compared to A549 cells treated with nicotine and the VEGF antibody (Figure 6C). Co-treatment of A549 cells with nicotine + 4-MU + VEGF antibody increased apoptosis ~3.95-fold, an effect more pronounced compared to A549 cells treated with nicotine and either 4-MU or the VEGF antibody. The largest increase in A549 cell apoptosis, ~5.88-fold, was observed in cells treated with nicotine + PKI + PFT-α + 4-MU + the VEGF antibody (Figure 6C). 

Treatment of H1299 cells with nicotine decreased apoptosis ~3.30-fold compared to control untreated cells (Figure 6D). In the presence of nicotine, H1299 cell apoptosis increased ~1.70-fold by co-incubation with PKI and ~2.40-fold by co-incubation with 4-MU, while no effects were found upon addition of PFT-α or hIgG control, compared to cells treated with only nicotine. Addition of the neutralizing VEGF antibodies increased H1299 cell apoptosis in the presence of nicotine ~1.85-fold compared to H1299 cells treated with nicotine and hIgG control (Figure 6D). 

Co-treatment of H1299 cells with nicotine + PKI + 4-MU increased apoptosis ~3.10-fold, an effect more pronounced compared to H1299 cells treated with nicotine and either PKI or 4-MU (Figure 6D). The effects observed by co-treatment of H1299 cells with nicotine + (PKI + PFT-α), (PFT-α + 4-MU), or (PFT-α + VEGF antibody) were indistinguishable from those in the absence of added PFT-α (Figure 6D). Co-treatment of H1299 cells with nicotine + PKI + VEGF antibody increased apoptosis ~3.00-fold compared to cells treated with only nicotine, an effect more pronounced compared to H1299 cells treated with nicotine and either PKI or the VEGF antibody. Co-treatment of H1299 cells with nicotine + 4-MU + VEGF antibody increased apoptosis ~3.13-fold compared to cells treated with only nicotine, an effect more pronounced compared to H1299 cells treated with nicotine and either 4-MU or the VEGF antibody. H1299 cell apoptosis increased ~4.45-fold in cells treated with nicotine + PKI + PFT-α + 4-MU + the VEGF antibody compared to cells treated with only nicotine (Figure 6D). 

In NSCLC, nicotine has been shown to increase HIF-1α and VEGF in A549 cells and enhance functional angiogenesis [58,59]. Conditioned media from nicotine-treated A549 cells was reported to enhance HUVEC tube formation in vitro and angiogenesis in vivo, and these effects diminished with pathway blockade, for example, by HIF-1α knockdown [58,59]. In xenografts, exposure to nicotine (with estradiol) increased A549 tumor VEGF levels and microvessel density, linking tumor-cell VEGF induction to angiogenesis in vivo [60]. Complementing these functional data, side-by-side A549 and H1299 experiments showed that nicotine increases VEGF in the conditioned media and that this response is suppressed by α-BTX, propranolol, and/or dihydro-β-erythroidine, indicating the potential control of nicotinic/β-adrenergic signaling on tumor-cell VEGF levels across NSCLC models [21]. This nicotine/VEGF/angiogenesis connection is also observed beyond NSCLC. For example, in gastric cancer, nicotine was shown to elevate cyclooxygenase-2 (COX-2) and VEGF and increase angiogenesis in sponge/Matrigel models, an effect reduced by COX-2 or VEGFR blockade [62]. In nasopharyngeal carcinoma, nicotine increased the VEGF/PEDF ratio through α7-nAChR, ERK, and HIF-1α signaling, consistent with a pro-angiogenic shift [20]. In esophageal cancer, nicotine was found to stabilize VEGF-C expression and promote lymphatic metastasis, underscoring nicotine-driven VEGF-family up-regulation in tumor biology [63]. Together, these studies demonstrate that nicotine-induced increases in tumor-cell VEGF are linked to functional angiogenesis in NSCLC, with parallel corroboration in other cancer systems.

In NSCLC, nicotine is known to promote long-term growth and clonogenicity while elevating pro-angiogenic and survival cues [59,60]. Nicotine has been reported to enhance clonogenic growth in NSCLC models, including A549 and H1299 cells [82]. Nicotine-responsive signals were also reported to drive long-term colony outgrowth in A549 cells [90]. Converging evidence also supports targeting nicotinic and/or adrenergic cAMP signaling in clonogenic assays. For example, β-adrenergic blockade (e.g., with propranolol) was reported to decrease A549/H1299 colony formation [91,92,93]. In NSCLC models, nicotine was reported to affect cAMP–PKA signaling, leading to growth and survival, supporting clonogenicity [30,94]. In A549 cells, nicotine activated PKA (via β-adrenergic signaling) and downstream survival nodes (e.g., Bad phosphorylation), placing PKA as regulator of nicotine signaling in lung cancer cells [28,30,53]. In H1299 and other NSCLC cell lines, nicotine rapidly increases p-CREB (a canonical PKA substrate), and this activation is β-blocker–sensitive, further linking receptor input to the cAMP–PKA axis [22,53,72,81,93,94]. PKA activity has also been linked to angiogenesis; for example, the PKA agonist 8-Br-cAMP was found to increase VEGF production in osteoblast-like MC3T3-E1 cells, promoting angiogenesis demonstrated by increased endothelial cell (HUVEC) migration and tubule formation [56]. Complementary NSCLC studies showed that cAMP/PKA-directed signaling promoted proliferation and CREB-dependent expression of genes (including the VEGF gene), consistent with a mechanism in which PKA activation facilitates long-term/colony outgrowth, a process attenuated by PKA blockade [28,33]. 

NSCLC cells show increased VEGF secretion after nicotine exposure, and nicotine has been shown to enhance tumor growth and angiogenesis in A549 xenografts, consistent with a VEGF-linked survival phenotype [5,26,58,59,60,62,63,95]. In NSCLC, clonogenic growth is known to be linked to VEGF signaling [96,97,98]. Direct VEGF knockdown was reported to reduce colony formation in lung cancer cells by combining VEGF siRNA with a PI3K/mTOR inhibitor that led to suppressed A549 and H460 colony formation and proliferation, indicating that VEGF is required for long-term outgrowth in vitro [96]. At the transcriptional level, downregulating VEGF-A (e.g., by miR-126 restoration) suppressed NSCLC cell growth, and VEGF/VEGFR-2 signaling upregulated enhancer of zeste homolog 2 (EZH2) to promote malignant phenotypes, mechanistic links consistent with VEGF supporting clonogenic survival [97,98]. 

Blocking HA signaling suppressed clonogenicity in lung cancer models, while CD44 overexpression was reported to augment colony formation in H1299 cells, whereas CD44 inhibition reduced proliferation and colony formation in NSCLC cells [99,100,101]. Incubation of cells with 4-MU reduced colony formation and viability in lung cancer models and across tumor types [99,100]. Parallel findings in other cancers showed that 4-MU suppressed HA and reduced anchorage-independent growth and colony formation, while nicotine promoted pro-angiogenic and proliferative programs, supporting the broader relevance of this mechanism while underscoring its preclinical nature [49,102]. 

In NSCLC, colony-forming capacity inversely correlates with functional p53 [87,103,104,105,106]. In A549 cells, activating or restoring p53 reduces long-term growth, whereas weakening p53 increases colony formation [87,104,106]. Activation of the p53 pathway by MDM2 antagonists (e.g., Nutlin-3a) has been reported to suppress proliferation and colony outgrowth in p53-WT lung cancer cells, and genetic manipulations that raise p53 activity similarly constrained clonogenicity [87,88,103,104,105,106]. Conversely, perturbations that dampen p53 (e.g., p53 depletion) increase A549 colony formation, consistent with a p53-dependent brake on clonogenic survival [103,104,105]. In H1299 cells, introducing wild-type p53 with tetracycline-regulated systems suppressed growth and colony formation, and oncogenic mutant p53 alleles were shown to enhance spheroid/colony phenotypes, underscoring that loss of p53 or gain-of-function mutant p53 favors clonogenic outgrowth [107,108,109]. Similar p53-dependent suppression of colony formation has been shown across other cancers (e.g., AML, sarcoma), where MDM2 inhibition activated p53 and reduced colonies, supporting the general role of p53 as a limiter of anchorage-independent and clonogenic growth [110].

### 2.7. 4-MU Reduces the Rescue of Cell Viability Resulting from Addition of Purified VEGF to Nicotine-Treated Cell Media Immunodepleted of Secreted VEGF

**Figure 7:**
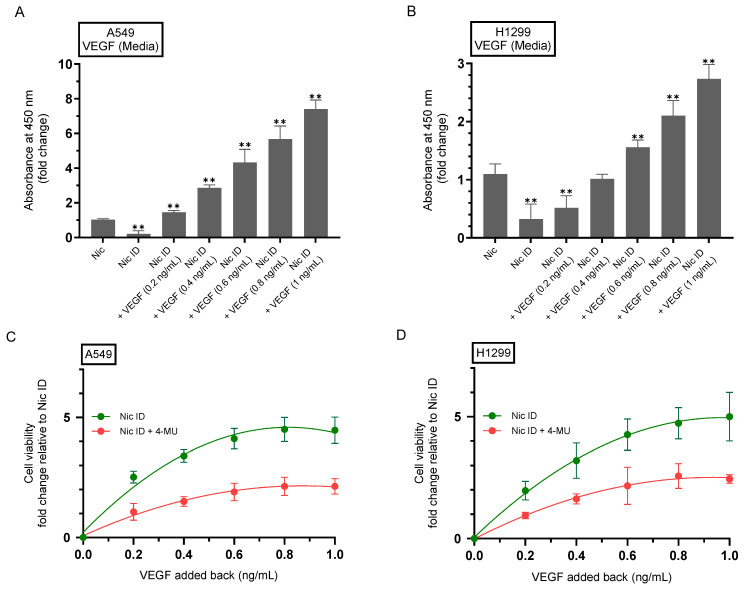
Cell viability was restored upon addition of purified VEGF to media from cells treated with nicotine and immunodepleted of secreted VEGF (Nic ID), an effect reduced in the presence of 4-MU. Cells were grown in media with 10% FBS for 24 h, serum-starved overnight, then incubated in serum-free media for 72 h in the presence of nicotine (Nic) (1 µM). The media from cells treated with nicotine was immunodepleted (ID) of secreted VEGF, then increasing concentrations of purified VEGF protein were added, followed by measurement of the levels of VEGF in A549 cell media (**A**) and H1299 cell media (**B**). Cell viability was measured as a function of increasing exogenously added VEGF to Nic ID media in the absence or presence of 4-MU (600 µM) (**C**,**D**). Data were averaged, normalized, and expressed relative to media of cells treated with nicotine (**A**,**B**) using the GraphPad 10.6.0 software. Data shown as mean ± SD; *n* = 5 biological replicates per time point. Technical replicates were averaged within each biological replicate before analysis. Asterisks indicate a statistically significant difference relative to Nic control, Mann–Whitney test, ** *p* < 0.01. Absence of asterisks indicates no significance.

Previously, we reported that nicotine elevates secreted VEGF and pro-survival signaling in NSCLC A549 and H1299 cells [21]. Under serum-free conditions, we found that anti-VEGF antibodies reduced viability and increased apoptosis with or without nicotine, and suppressed PI3K/AKT/NFκB activity [21]. Moreover, VEGF levels in the conditioned media increased with nicotine and decreased when VEGF was neutralized or when cholinergic/adrenergic inputs were blocked, findings that support a VEGF-dependent survival axis in both A549 and H1299 cells [21]. Independent NSCLC studies corroborate an autocrine/paracrine VEGF requirement, as neutralizing VEGF diminished proliferation in VEGF-responsive lung cancer cells, including A549 [89]. In this study, we immunodepleted VEGF from nicotine-conditioned media to determine whether the increase in cell viability observed upon treatment of A549 cells with nicotine (Figure 6A) and upon treatment of H1299 cells with nicotine (Figure 6B) depends, in part, on the VEGF component of the secretome. Immunodepletion of VEGF from nicotine-treated A549 and H1299 cell media (Methods) was effective in decreasing the levels of VEGF in the media of both cell lines (Figure 7A,B). As expected, addition of increasing concentrations of purified VEGF to the media led to a corresponding rise in its detection (Figure 7A,B). We also tested whether cell viability of A549 and H1299 cells immunodepleted of VEGF can be rescued by the addition of purified VEGF to the media of both cell lines (Figure 7C,D). For the cell viability assays, we used 4-MU in parallel (Figure 7C,D) to inhibit HA–CD44 signaling known to be implicated in NSCLC growth and modulating the PI3K/AKT pathways that intersect with VEGF signaling [100,111,112]. Our results show that 4-MU was able to attenuate the maximal rescue of cell viability observed in the absence of 4-MU even at the highest concentrations of VEGF used for both cell lines (Figure 7C,D). 

## 3. Conclusions

**Figure 8:**
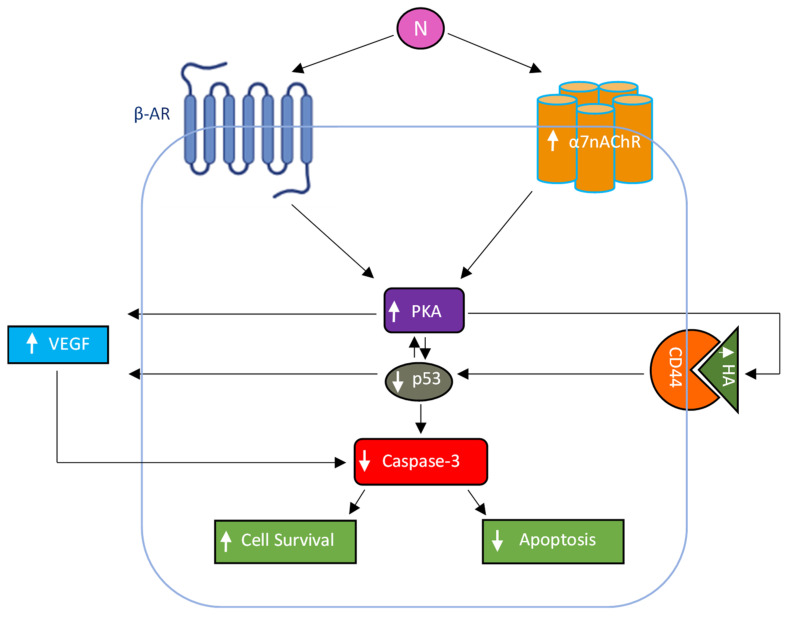
Summary of the main findings of this study. Nicotine acts via either the nicotinic acetylcholine receptor. (α7nAChR), or the β-Adrenergic receptors (β-ARs), activates PKA, increasing HA levels and activation of HA-CD44 signaling, inhibiting p53, increasing the levels of VEGF in the media, leading to increased cell survival and decreased apoptosis.

This study demonstrates a novel role of PKA in regulating the levels of VEGF in the conditioned media of NSCLC cells in the absence or presence of nicotine. VEGF levels decreased upon inhibition of PKA or HA synthesis in both A549 and H1299 cells but increased when p53 activity was blocked in A549 cells, revealing a novel crosstalk between PKA, HA, and p53 in controlling VEGF levels. Nicotine-induced PKA activation was diminished by co-treatment with α-Btx and propranolol, linking cholinergic and adrenergic signaling to VEGF regulation. Moreover, knockdown of p53 increased PKA activity and VEGF levels in the conditioned media, further highlighting the antagonistic relationship between these pathways. Functionally, co-treatment with nicotine and PKI, 4-MU, or VEGF antibodies reduced cell viability and increased apoptosis, indicating that targeting PKA-dependent VEGF regulation may sensitize NSCLC cells to growth inhibition. Our findings are summarized in Figure 8. A limitation of this study is that the findings are restricted to two NSCLC cell lines and rely on in vitro assays; thus, further validation in additional models and in vivo systems will be necessary to confirm the broader relevance of the PKA–HA–p53 axis in nicotine-driven NSCLC progression.

Our in vitro nicotine doses were selected to be physiologically relevant to airway and tumor microenvironments [113,114,115]. In smokers and vapers, plasma nicotine typically peaks around 10–30 ng/mL (~0.06–0.19 µM) and declines with a ~2 h half-life, with higher peaks achievable in some e-cigarette conditions [116,117]. Nicotine levels in airway surface fluid (~0.5–5 µM) fall within the typical ranges (0.1–1 µM) used in A549/H1299 cell studies [113,114,115,118]. Thus, our nicotine exposure conditions model upper-airway and tumor-adjacent levels rather than average plasma concentrations and should be interpreted as preclinical, not clinical, dosing recommendations.

In NSCLC, nicotine-induced VEGF upregulation and survival signaling are well-documented in A549 and H1299 cells [5,21,58,59,60] and align with our findings from this study. Our data extend this literature by positioning PKA, HA–CD44, and p53 as modulators of VEGF levels and secretion in A549 and H1299 cells. Collectively, our findings offer mechanistic support for the role of the PKA–HA–p53 axis in regulating nicotine-induced VEGF levels in the conditioned media of NSCLC in vitro, but further in vivo validation is needed.

## 4. Materials and Methods

### 4.1. Materials

Most of the material used in this study was purchased, as we reported previously [75,112,119,120,121,122]. Phosphate-buffered saline (PBS), nitrocellulose membranes, nicotine, 4-methylumbelliferone (4-MU, M1381), pifithrin-α, epinephrine, propranolol hydrochloride, nutlin-3a, and α-bungarotoxin (α-BTX) were purchased from Sigma-Aldrich. Halt protease and phosphatase inhibitor cocktail, BCA protein assay kit, super signal west pico luminol (chemiluminescence) reagent, α-tubulin mouse monoclonal antibody (DM1A), goat anti-mouse IgG (H + L) superclonal secondary antibody, HRP conjugate (A28177), 3,3’,5,5’-tetramethylbenzidine (TMB), lipofectamine 2000 transfection reagent, human IgG (hIgG) isotype control, and goat anti-rabbit IgG (H + L) secondary antibody (HRP, 31466) were purchased from ThermoFisher. SignalSilence p53 siRNA I (6231), SignalSilence control siRNA (unconjugated, 6568), PKA C-α (D38C6) rabbit mAb #5842, and p53 rabbit antibody (9282) were purchased from Cell Signaling Technology. Human cleaved caspase-3 (Asp175) ELISA kit, anti-MDM2 rabbit antibody [EPR22256-98], and donkey anti-mouse IgG (HRP) (ab205724) were purchased from Abcam. Human/primate VEGF antibody (MAB293-100), PKI 14-22 amide, myristoylated (2546), recombinant human VEGF 165 protein (293-VE), and 8-Bromo-cAMP, sodium salt (1140), were purchased from R&D Systems. MDM2 antibody (SMP14): sc-965, p53 antibody (mouse, DO-1): sc-126, PKA IIα reg (H-12) mouse mAb, sc-137220 were from Santa Cruz. 

### 4.2. Cell Culture

Human NSCLC cell lines, A549 (ATCC CCL-185) and H1299 (ATCC CRL-5803), were obtained from the American Type Culture Collection (ATCC, Manassas, VA, USA). Cells were cultured following our previously reported protocols [75,119,120,121,123,124] in DMEM/F12 media in the presence of 10% fetal bovine serum (FBS), 50 U/mL penicillin, and 50 U/mL streptomycin at 37 °C, 95% humidity, and 5% CO_2_. Cells were counted with a hemocytometer after trypan blue staining. A549 and H1299 cell lines were passaged upon reaching 80–90% confluence using standard trypsinization protocols, with dilutions ranging from approximately 1:5 to 1:10. Cultures were maintained for a period of up to three weeks. After thawing, the passage number was maintained at 20 or below. Cells were routinely monitored for morphological characteristics and p53 status through ELISA and Western blot analysis. In certain experiments, cells were maintained in culture for 24, 48, and 72 h. A 72 h incubation period was used in cell culture experiments examining secretomes, as the extended duration facilitates higher accumulation and improved detection of secreted factors in the conditioned media.

### 4.3. MTT Assay

Cell viability was assessed in 96-well plates using the MTT reduction assay (Sigma-Aldrich, Saint Louis, MO, USA), as we previously described [34,35,112,122,125]. Viable cells have the ability to convert MTT into purple formazan crystals. Wells with untreated cells served as positive controls, while wells containing only DMSO and cell-free culture media served as negative controls. Following incubation, formazan crystals were dissolved, and absorbance was measured at 570 nm using a plate reader. The results were normalized to cell number (absorbance/cell number). All absorbance readings were within the linear range. Statistical analysis was carried out with GraphPad Prism version 10.6.0 for Windows. 

### 4.4. Caspase-3 Assay 

Cleaved caspase-3 levels were quantified using the Human Cleaved Caspase-3 (Asp175) ELISA Kit (Abcam, ab220655, Waltham, MA, USA) following the manufacturer’s protocol. This assay employs a capture antibody and detector antibody complex that binds the target in solution and is immobilized via an anti-tag antibody pre-coating the well. Briefly, A549 and H1299 cells were harvested after treatment, lysed in the supplied extraction buffer, and clarified by centrifugation at 13,000× *g* for 10 min at 4 °C. Equal amounts of total protein from each lysate were loaded into 96-well plates pre-coated with an antibody specific for cleaved caspase-3. After incubation with the capture antibody complex, wells were washed, and detection was performed using HRP-conjugated secondary antibody and TMB substrate. Absorbance was measured at 450 nm using a microplate reader. Concentrations were determined from a standard curve generated using recombinant cleaved caspase-3 provided with the kit. Negative controls included buffer-only blanks and media not incubated with cells. Positive controls included the recombinant cleaved caspase-3 standard. Data were normalized to total protein concentration determined by the BCA assay and expressed relative to untreated controls.

### 4.5. p53 Transcription Factor Activity Assay

The p53 activity was assayed using the human p53 transcription factor activity assay kit (RayBio, TFEH-p53, Peachtree Corners, GA, USA), as we reported earlier [35,112,122,126]. Double-stranded oligonucleotides with the p53 binding sequence were immobilized on 96-well plates to capture active p53 from cell lysates. Briefly, lysates were prepared from A549 and H1299 cells harvested under the indicated conditions. Equal concentrations of protein were incubated in 96-well plates pre-coated with a double-stranded DNA consensus sequence specific for the p53 binding site. Activated p53 within the cell extracts interacted with the immobilized oligonucleotides, and the resulting complex was subsequently identified using a p53-specific primary antibody followed by an HRP-conjugated secondary antibody. Colorimetric detection was carried out using a TMB substrate, and absorbance was measured at 450 nm with a microplate reader. The activity of p53 was expressed as relative absorbance units normalized to protein concentration, with A549 extracts serving as a positive control and H1299 extracts (p53-null) serving as a negative control.

### 4.6. Quantitation of Protein Levels and Normalization to α-Tubulin

The Human p53 ELISA Kit (ab171571) was used to quantitate total p53 levels. In brief, lysates from A549 and H1299 cells were prepared using the provided lysis buffer and clarified by centrifugation. Equal amounts of total protein were subsequently added to 96-well plates pre-coated with an anti-p53 capture antibody. Bound p53 was detected using a biotinylated detection antibody and an HRP-streptavidin conjugate, followed by development with TMB substrate. Absorbance at 450 nm was measured, and p53 concentrations were determined from a standard curve with recombinant p53 protein, then normalized to total protein concentration. Quantification of α-tubulin was performed using the human α-tubulin ELISA kit (A312476). Cell lysates were added to wells pre-coated with an α-tubulin capture antibody. Detection of the bound protein was carried out using an HRP-conjugated secondary antibody followed by TMB substrate development. Absorbance at 450 nm was measured, and concentrations were determined using a recombinant α-tubulin standard curve. Once the concentrations of p53 and α-tubulin in each sample were quantitated using their respective ELISA standard curves, the ratios of total p53 to α-tubulin were then calculated and plotted for each sample.

### 4.7. VEGF Concentration Determination

The concentration of VEGF in the cell culture media was measured as we reported earlier [21] using the human VEGF solid-phase sandwich ELISA kit (ThermoFisher, KHG0111, Waltham, MA, USA). The conditioned media obtained from A549 and H1299 cells was added into 96-well plates that had been pre-coated with a VEGF-specific capture antibody. Following incubation and subsequent washing steps, a secondary biotinylated anti-VEGF detection antibody was added, after which streptavidin-HRP and the TMB substrate were sequentially added. Absorbance was determined at 450 nm following the addition of streptavidin-HRP and the substrate solution. VEGF levels were measured using a standard curve prepared with recombinant human VEGF. Results were normalized to total protein concentration. The intensity of the signal was proportional to the concentration of VEGF in the media. Assay buffer alone was used as a negative control, and recombinant human VEGF was used as a positive control. The human VEGF-A cell lysates ELISA kit (ThermoFisher, EHVEGFACL, Waltham, MA, USA) was used to measure the intracellular VEGF levels according to the instructions provided by the manufacturer. 

### 4.8. PKA Assay

PKA activity was measured using the solid-phase PKA Activity Assay Kit (Invitrogen, EIAPKA, Carlsbad, CA, USA) following the manufacturer’s instructions. Briefly, two antibodies are used to detect the substrate phosphorylated by PKA in the presence of ATP. Cell lysates were prepared in kinase extraction buffer, and equal amounts of protein were added to wells coated with a PKA-specific substrate peptide. Active PKA phosphorylated the immobilized substrate, which was subsequently detected using a phospho-specific antibody followed by an HRP-conjugated secondary antibody. The signal was developed with TMB substrate, and absorbance was measured at 450 nm using a microplate reader. PKA activity was quantified by using a calibration curve generated with recombinant active PKA and normalized to total protein concentration. Positive controls included recombinant active PKA supplied with the kit, and negative controls included blank wells (buffer only and media not incubated with cells).

### 4.9. HA Quantitation

HA levels were measured using the hyaluronan quantikine ELISA kit (R&D Systems, DHYAL0, Minneapolis, MN, USA) following the manufacturer’s protocol. Briefly, conditioned media was collected, clarified by centrifugation, and added to 96-well microplates pre-coated with a capture antibody specific for HA. After washing, a biotinylated detection reagent was applied, followed by streptavidin–HRP and the TMB substrate. The reaction was stopped with sulfuric acid solution, and absorbance was measured at 450 nm with wavelength correction at 570 nm. HA concentrations were calculated by interpolation from a standard curve generated using purified HA standards supplied with the kit. Data were normalized to viable cell number or total protein to account for variation between samples. HA standards provided in the kit were used as positive controls, and wells with media not incubated with cells and those incubated with buffer only were used as negative controls. 

### 4.10. Western Blotting

Cell lysates were collected as indicated and analyzed according to our earlier methods [34,75,112,119,122,124,125,126]. Samples were separated using 12% SDS-PAGE and transferred to a nitrocellulose membrane. After blocking with 5% nonfat milk in TBST and performing washing steps, the membrane was incubated overnight at 4 °C with primary antibodies directed against p53 or α-tubulin. The membrane was washed and incubated with HRP-conjugated secondary antibodies diluted according to the manufacturer’s instructions for 1 h at room temperature (RT). After additional washing, the blots were developed using SuperSignal West Pico luminol chemiluminescent reagent and imaged with a Bio-Rad molecular imager. The blot was subsequently stripped and reprobed using Restore Western Blot Stripping Buffer (Thermo Fisher, Waltham, MA, USA) as directed by the manufacturer. Signals for p53 were normalized to α-tubulin. Western blot band intensities were quantified by densitometry using ImageJ v1.54r (NIH), with background subtraction, see Supplementary Material.

### 4.11. siRNA Transfection

Transfections were carried out according to our earlier methods [35,75,122,124,125,126]. Control siRNA or p53 siRNA were each mixed with Lipofectamine 2000 transfection reagent diluted in Opti-MEM Reduced Serum Media (ThermoFisher, Waltham, MA, USA) and incubated at RT for 20 min to allow complex formation. The mixtures were subsequently added to the cells and incubated for 12 h at 37 °C, after which the specified treatments were applied. Each measurement is presented as the mean ± S.D. from three to five independent experiments, each conducted in at least triplicate. Knockdown efficiency was assessed using ELISAs and/or Western blotting.

### 4.12. Immunodepletion

Conditioned media were immunodepleted as described in our previous reports [35,112,121,122,123,124]. Briefly, media were incubated with anti-VEGF antibody (20 µg/mL) or, as a negative control, human isotype IgG (20 µg/mL). After removal of the antibody complex, the resulting immunodepleted supernatants were collected, and the residual VEGF was quantified by ELISA (Thermo Fisher, Waltham, MA, USA) to verify depletion prior to use.

### 4.13. Statistical Analysis

The analysis was performed as we previously reported [34,75,112,120,121,122,124,125,126]. Analyses were performed in GraphPad Prism (v10.6.0). Unless noted, tests were two-tailed with α = 0.05, and data are shown as mean ± SD. For comparisons of ≥3 groups within a cell line/assay, we used one-way ANOVA with Dunnett’s multiple comparisons (vs. a prespecified control, e.g., Control or Nicotine) or Tukey’s (all pairs). For two-group contrasts, we used an unpaired *t*-test; when assumptions were not met, we used Mann–Whitney. For time- or dose-series, we used two-way ANOVA (or a mixed-effects model if there were missing values and/or repeated measures). We first tested for overall differences between groups and/or over time and then performed pairwise comparisons with correction for multiple testing (Šidák or Dunnett, as appropriate). Assumption checks included Shapiro–Wilk (normality, with Q–Q inspection) and Brown–Forsythe/Levene (variance homogeneity). When assumptions were doubtful, we used Welch’s ANOVA (unequal variances) or Kruskal–Wallis with Dunn’s post hoc. We report exact *p*-values; when multiple comparisons were performed, we report the adjusted *p*-values. “*n*” denotes biological replicates; where technical replicates were collected (e.g., duplicate ELISA wells), they were averaged within each biological replicate before analysis. Effect size is reported as fold change ± SD relative to the stated reference group.

## Figures and Tables

**Table 1:** Summary of VEGF levels in A549 and H1299 cell media.

A549 Cell Summary—VEGF Levels		
Condition (72 h)	VEGF Level Fold Change ± SD	*p*-value
Control	VEGF levels increased over time	
8-Br-cAMP	~1.85-fold ± 0.22 ↑ vs. control	0.017
PKI	~1.45-fold ± 0.17 ↓ vs. control	0.026
Nicotine	~2.45-fold ± 0.28 ↑ vs. control	0.022
Nicotine + 8-Br-cAMP	~1.25-fold ± 0.15 ↑ vs. nicotine	0.018
Nicotine + PKI	~1.45-fold ± 0.16↓ vs. nicotine	0.009
**H1299 Cell Summary–VEGF Levels**		
Condition (72 h)	VEGF Level Fold Change ± SD	*p*-value
Control	VEGF levels increased over time	
8-Br-cAMP	~1.40-fold ± 0.16 ↑ vs. control	0.027
PKI	~1.60-fold ± 0.19 ↓ vs. control	0.013
Nicotine	~2.65-fold ± 0.30 ↑ vs. control	0.007
Nicotine + 8-Br-cAMP	~1.20-fold ± 0.14 ↑ vs. nicotine	0.024
Nicotine + PKI	~1.60-fold ± 0.20 ↓ vs. nicotine	0.004

**Table 2:** Summary of VEGF levels using different conditions.

A549 Cell Summary		
Condition	VEGF Fold Change ± SD	*p*-value
Nicotine + PKI	~1.40-fold ± 0.16 ↓ vs. nicotine	0.009
Nicotine + 4-MU	~1.40-fold ± 0.14 ↓ vs. nicotine	0.008
Nicotine + PKI + 4-MU	~2.00-fold ± 0.23 ↓ vs. nicotine	0.007
Nicotine + PFT-α	~1.50-fold ± 0.42 ↑ vs. nicotine	0.009
Nicotine + PKI + PFT-α	~1.40-fold ± 0.16 ↓ vs. nicotine + PFT-α	0.008
Nicotine + 4-MU + PFT-α	~1.40-fold ± 0.17 ↓ vs. nicotine + PFT-α	0.008
Nicotine + PKI + 4-MU + PFT-α	~2.00-fold ± 0.24 ↓ vs. nicotine + PFT-α	0.009
**H1299 Cell Summary**		
Condition	VEGF Fold Change ± SD	*p*-value
Nicotine + PKI	~1.55-fold ± 0.18 ↓ vs. nicotine	0.008
Nicotine + 4-MU	~1.55-fold ± 0.19 ↓ vs. nicotine	0.006
Nicotine + PKI + 4-MU	~2.00-fold ± 0.23 ↓ vs. nicotine	0.003
Nicotine + PFT-α	No effect (p53-null) vs. nicotine	n.s.
Nicotine + PKI + PFT-α	No effect vs. nicotine + PKI	n.s.
Nicotine + 4-MU + PFT-α	No effect vs. nicotine + 4-MU	n.s.
Nicotine + PKI + 4-MU + PFT-α	No effect vs. nicotine + PKI + 4-MU	n.s.

**Table 3:** Summary of PKA Activity and HA Levels Under Various Treatments.

A549 Cell Summary—PKA Activity		
Condition	PKA Activity Fold Change ± SD	*p*-value
Epinephrine	~2.85-fold ± 0.33 ↑ vs. control	0.003
Epinephrine + Propranolol	Blocked effect	n.s.
Nicotine	~2.55-fold ± 0.29 ↑ vs. control	0.002
Nicotine + α-Btx	~1.40-fold ± 0.16 ↓ vs. nicotine	0.004
Nicotine + Propranolol	~1.25-fold ± 0.14 ↓ vs. nicotine	0.003
Nicotine + α-Btx + Propranolol	~2.00-fold ± 0.22 ↓ vs. nicotine	0.004
PKI	Blocked activity	n.s.
**H1299 Cell Summary—PKA Activity**		
Condition	PKA Activity Fold Change ± SD	*p*-value
Epinephrine	~3.50-fold ± 0.40 ↑vs. control	0.001
Epinephrine + Propranolol	Blocked effect	n.s.
Nicotine	~3.15-fold ± 0.35 ↑ vs. control	0.005
Nicotine + α-Btx	~1.35-fold ± 0.15 ↓ vs. nicotine	0.003
Nicotine + Propranolol	~1.20-fold ± 0.14 ↓ vs. nicotine	0.002
Nicotine + α-Btx + Propranolol	~1.70-fold ± 0.19 ↓ vs. nicotine	0.005
PKI	Blocked activity	n.s.
**A549 Cell Summary—HA Levels**		
Condition	HA Level Fold Change ± SD	*p*-value
Epinephrine	~2.20-fold ± 0.25 ↑ vs. control	0.004
Epinephrine + Propranolol	Blocked effect	n.s.
Nicotine	~2.00-fold ± 0.22 ↑ vs. control	0.006
Nicotine + α-Btx	~1.50-fold ± 0.19 ↓ vs. nicotine	0.003
Nicotine + Propranolol	~1.20-fold ± 0.15 ↓ vs. nicotine	0.002
Nicotine + α-Btx + Propranolol	~1.85-fold ± 0.21 ↓ vs. nicotine	0.004
PKI (no nicotine)	~2.30-fold ± 0.26 ↓ vs. control	0.003
Nicotine + PKI	~1.50-fold ± 0.17 ↑ vs. control + PKI	0.007
Nicotine + PKI + α-Btx	~1.28-fold ± 0.15 ↓ vs. nicotine + PKI	0.008
Nicotine + PKI + Propranolol	~1.05-fold ± 0.12 ↓ vs. nicotine + PKI	0.007
Nicotine + PKI + α-Btx + Propranolol	~1.34-fold ± 0.15 ↓ vs. nicotine + PKI	0.006
**H1299 Cell Summary—HA Levels**		
Condition	HA Level Fold Change ± SD	*p*-value
Epinephrine	~2.65-fold ± 0.30 ↑ vs. control	0.005
Epinephrine + Propranolol	Blocked effect	n.s.
Nicotine	~2.30-fold ± 0.26 ↑ vs. control	0.006
Nicotine + α-Btx	~1.48-fold ± 0.17 ↓ vs. nicotine	0.003
Nicotine + Propranolol	~1.25-fold ± 0.14 ↓ vs. nicotine	0.001
Nicotine + α-Btx + Propranolol	~1.75-fold ± 0.19 ↓ vs. nicotine	0.004
PKI (no nicotine)	~1.95-fold ± 0.22 ↓ vs. control	0.006
Nicotine + PKI	~1.45-fold ± 0.16 ↑ vs. control + PKI	0.031
Nicotine + PKI + α-Btx	~1.30-fold ± 0.15 ↓ vs. nicotine + PKI	0.003
Nicotine + PKI + Propranolol	~1.08-fold ± 0.13 ↓ vs. nicotine + PKI	0.002
Nicotine + PKI + α-Btx + Propranolol	~1.40-fold ± 0.16 ↓ vs. nicotine + PKI	0.006

**Table 4:** Summary of p53 activity and VEGF levels under various treatments.

A549 Cell Summary—p53 Activity		
Condition	p53 Activity Fold Change ± SD	*p*-value
Epinephrine	~1.40-fold ± 0.16 ↓ vs. control	0.004
Epinephrine + Propranolol	Abolished effect	n.s.
Nicotine	~2.40-fold ± 0.27 ↓ vs. control	0.008
Nicotine + α-Btx	~1.60-fold ± 0.18 ↑ vs. nicotine	0.007
Nicotine + Propranolol	~1.60-fold ± 0.19 ↑ vs. nicotine	0.002
Nicotine + α-Btx + Propranolol	~1.95-fold ± 0.22 ↑ vs. nicotine	0.003
PKI (no nicotine)	~1.75-fold ± 0.2 ↑ vs. control	0.006
Nicotine + PKI	~2.20-fold ± 0.25 ↑ vs. nicotine	0.004
Nicotine + PKI + α-Btx	~1.40-fold ± 0.15 ↑ vs. nicotine + PKI	0.006
Nicotine + PKI + Propranolol	~1.35-fold ± 0.15 ↑ vs. nicotine + PKI	0.003
Nicotine + PKI + α-Btx + Propranolol	~1.65-fold ± 0.19 ↑ vs. nicotine + PKI	0.001
**A549 Cell Summary—VEGF Levels**		
Condition	VEGF Level Fold Change ± SD	*p*-value
Epinephrine	~1.50-fold ± 0.170 ↑ vs. control	0.004
Epinephrine + Propranolol	Abolished effect	n.s.
Nicotine	~2.55-fold ± 0.29 ↑ vs. control	0.006
Nicotine + α-Btx	~1.55-fold ± 0.17 ↓ vs. nicotine	0.004
Nicotine + Propranolol	~1.30-fold ± 0.15 ↓ vs. nicotine	0.006
Nicotine + α-Btx + Propranolol	~1.85-fold ± 0.21 ↓ vs. nicotine	0.007
PKI (no nicotine)	~1.40-fold ± 0.15 ↓ vs. control	0.005
Nicotine + PKI	~1.45-fold ± 0.16 ↓ vs. nicotine	0.004
Nicotine + PKI + α-Btx	~1.65-fold ± 0.19 ↓ vs. nicotine + PKI	0.030
Nicotine + PKI + Propranolol	~1.35-fold ± 0.16 ↓ vs. nicotine + PKI	0.040
Nicotine + PKI + α-Btx + Propranolol	~2.20-fold ± 0.26 ↓ vs. nicotine + PKI	0.003
**H1299 Cell Summary—VEGF Levels**		
Condition	VEGF Level Fold Change ± SD	*p*-value
Epinephrine	~1.70-fold ± 0.19 ↑ vs. control	0.007
Epinephrine + Propranolol	Blocked effect	n.s.
Nicotine	~2.70-fold ± 0.32 ↑ vs. control	0.002
Nicotine + α-Btx	~1.45-fold ± 0.16 ↓ vs. nicotine	0.005
Nicotine + Propranolol	~1.30-fold ± 0.15 ↓ vs. nicotine	0.003
Nicotine + α-Btx + Propranolol	~1.70-fold ± 0.20 ↓ vs. nicotine	0.004
PKI (no nicotine)	~1.60-fold ± 0.19 ↓ vs. control	0.003
Nicotine + PKI	~1.70-fold ± 0.21 ↓ vs. nicotine	0.002
Nicotine + PKI + α-Btx	~1.40-fold ± 0.16 ↓ vs. nicotine + PKI	0.030
Nicotine + PKI + Propranolol	~1.30-fold ± 0.15 ↓ vs. nicotine + PKI	0.026
Nicotine + PKI + α-Btx + Propranolol	~1.70-fold ± 0.20 ↓ vs. nicotine + PKI	0.003

**Table 5:** Summary of PKA activity and VEGF levels with p53 siRNA.

A549 Cell Summary—PKA Activity		
Condition	PKA Activity Fold Change ± SD	*p*-value
p53 siRNA (untreated)	~1.35-fold ± 0.15 ↑ vs. control siRNA	0.004
p53 siRNA + Nicotine	~1.30-fold ± 0.15 ↑ vs. control siRNA + Nicotine	0.002
H1299 (any condition)	No effect (p53-null)	n.s.
**A549 Cell Summary—VEGF Levels**		
Condition	VEGF Level Fold Change ± SD	*p*-value
p53 siRNA (untreated)	~2.00-fold ± 0.22 ↑ vs. control siRNA	0.006
p53 siRNA + Nicotine	~2.00-fold ± 0.23 ↑ vs. control siRNA + Nicotine	0.002
Control siRNA + PKI	~1.45-fold ± 0.16 ↓ vs. control siRNA (no PKI)	0.027
p53 siRNA + PKI	~1.55-fold ± 0.18 ↓ vs. p53 siRNA (no PKI)	0.004
Control siRNA + Nicotine + PKI	~1.45-fold ± 0.17 ↓ vs. control siRNA + Nicotine (no PKI)	0.031
p53 siRNA + Nicotine + PKI	~1.65-fold ± 0.19 ↓ vs. p53 siRNA + Nicotine (no PKI)	0.005
**H1299 Cell Summary—VEGF Levels**		
Condition	VEGF Level Fold Change ± SD	*p*-value
Nicotine (control or p53 siRNA)	~2.70-fold ± 0.31 ↑ vs. untreated	0.006
Control siRNA + PKI	~1.55-fold ± 0.18 ↓ vs. control siRNA (no PKI)	0.039
p53 siRNA + PKI	~1.55-fold ± 0.19 ↓ vs. p53 siRNA (no PKI)	0.040
Control siRNA + Nicotine + PKI	~1.60-fold ± 0.18 ↓ vs. control siRNA + Nicotine (no PKI)	0.004
p53 siRNA + Nicotine + PKI	~1.60-fold ± 0.19 ↓ vs. p53 siRNA + Nicotine (no PKI)	0.005

**Table 6:** Summary of cell viability and apoptosis with nicotine and inhibitors.

A549 Cell Summary—Cell Viability		
Condition	Cell Viability Fold Change ± SD	*p*-value
Nicotine	~1.75-fold ± 0.20 ↑ vs. control	0.002
Nicotine + PKI	~1.53-fold ± 0.17 ↓ vs. nicotine	0.006
Nicotine + 4-MU	~2.00-fold ± 0.24 ↓ vs. nicotine	0.003
Nicotine + PFT-α	~1.30-fold ± 0.15 ↑ vs. nicotine	0.005
Nicotine + VEGF antibody	~1.53-fold ± 0.18 ↓ vs. nicotine + hIgG	0.004
Nicotine + PKI + 4-MU	~3.30-fold ± 0.38 ↓ vs. nicotine	0.005
Nicotine + PKI + PFT-α	~1.23-fold ± 0.14 ↓ vs. nicotine	0.008
Nicotine + PFT-α + 4-MU	~1.60-fold ± 0.19 ↓ vs. nicotine	0.006
Nicotine + PKI + VEGF antibody	~2.65-fold ± 0.32 ↓ vs. nicotine	0.002
Nicotine + PFT-α + VEGF antibody	~1.23-fold ± 0.15 ↓ vs. nicotine	0.008
Nicotine + 4-MU + VEGF antibody	~3.05-fold ± 0.36 ↓ vs. nicotine	0.003
Nicotine + PKI + PFT-α + 4-MU + VEGF antibody	~4.66-fold ± 0.52 ↓ vs. nicotine	0.002
**H1299 Cell Summary—Cell Viability**		
Condition	Cell Viability Fold Change ± SD	*p*-value
Nicotine	~2.80-fold ± 0.32 ↑ vs. control	0.002
Nicotine + PKI	~1.43-fold ± 0.17 ↓ vs. nicotine	0.008
Nicotine + 4-MU	~1.58-fold ± 0.19 ↓ vs. nicotine	0.007
Nicotine + PFT-α	No effect (p53-null) vs. nicotine	n.s.
Nicotine + hIgG	No effect vs. nicotine	n.s.
Nicotine + VEGF antibody	~1.38-fold ± 0.16 ↓ vs. nicotine + hIgG	0.004
Nicotine + PKI + 4-MU	~2.28-fold ± 0.27 ↓ vs. nicotine	0.003
Nicotine + PKI + PFT-α	No effect (p53-null) vs. nicotine + PKI	n.s.
Nicotine + PFT-α + 4-MU	No effect (p53-null) vs. nicotine + 4-MU	n.s.
Nicotine + PFT-α + VEGF antibody	No effect (p53-null) vs. nicotine + VEGF antibody	n.s.
Nicotine + PKI + VEGF antibody	~2.10-fold ± 0.24 ↓ vs. nicotine	0.004
Nicotine + 4-MU + VEGF antibody	~2.26-fold ± 0.27 ↓ vs. nicotine	0.006
Nicotine + PKI + PFT-α + 4-MU + VEGF antibody	~2.58-fold ± 0.30 ↓ vs. nicotine	0.004
**A549 Cell Summary—Apoptosis**		
Condition	Apoptosis Fold Change ± SD	*p*-value
Nicotine	~2.50-fold ± 0.30 ↓ vs. control	0.002
Nicotine + PKI	~2.10-fold ± 0.24 ↑ vs. nicotine	0.006
Nicotine + 4-MU	~2.93-fold ± 0.33 ↑ vs. nicotine	0.003
Nicotine + PFT-α	~2.00-fold ± 0.23 ↓ vs. nicotine	0.004
Nicotine + VEGF antibody	~2.10-fold ± 0.24 ↑ vs. nicotine + hIgG	0.005
Nicotine + PKI + 4-MU	~4.00-fold ± 0.46 ↑ vs. nicotine	0.004
Nicotine + PKI + PFT-α	~1.58-fold ± 0.18 ↑ vs. nicotine	0.006
Nicotine + PFT-α + 4-MU	~2.15-fold ± 0.25 ↑ vs. nicotine	0.007
Nicotine + PKI + VEGF antibody	~3.80-fold ± 0.45 ↑ vs. nicotine	0.002
Nicotine + PFT-α + VEGF antibody	~1.68-fold ± 0.20 ↑ vs. nicotine	0.008
Nicotine + 4-MU + VEGF antibody	~3.95-fold ± 0.47 ↑ vs. nicotine	0.002
Nicotine + PKI + PFT-α + 4-MU + VEGF antibody	~5.88-fold ± 0.70 ↑ vs. nicotine	0.003
**H1299 Cell Summary—Apoptosis**		
Condition	Apoptosis Fold Change ± SD	*p*-value
Nicotine	~3.30-fold ± 0.38 ↓ vs. control	0.003
Nicotine + PKI	~1.70-fold ± 0.20 ↑ vs. nicotine	0.005
Nicotine + 4-MU	~2.40-fold ± 0.27 ↑ vs. nicotine	0.004
Nicotine + PFT-α	No effect (p53-null) vs. nicotine	n.s.
Nicotine + hIgG	No effect vs. nicotine	n.s.
Nicotine + VEGF antibody	~1.85-fold ± 0.22 ↑ vs. nicotine + hIgG	0.007
Nicotine + PKI + 4-MU	~3.10-fold ± 0.36 ↑ vs. nicotine	0.004
Nicotine + PKI + PFT-α	No effect (p53-null) vs. nicotine + PKI	n.s.
Nicotine + PFT-α + 4-MU	No effect (p53-null) vs. nicotine + 4-MU	n.s.
Nicotine + PFT-α + VEGF antibody	No effect (p53-null) vs. nicotine + VEGF antibody	n.s.
Nicotine + PKI + VEGF antibody	~3.00-fold ± 0.34 ↑ vs. nicotine	0.002
Nicotine + 4-MU + VEGF antibody	~3.13-fold ± 0.37 ↑ vs. nicotine	0.006
Nicotine + PKI + PFT-α + 4-MU + VEGF antibody	~4.45-fold ± 0.51 ↑ vs. nicotine	0.003

## Data Availability

The original contributions presented in this study are included in the article/Supplementary Material. Further inquiries can be directed to the corresponding author.

## Supplementary Materials

The following supporting information can be downloaded at: https://www.mdpi.com/article/10.3390/ijms262211103/s1.
