# Supplementary material for: Nicotine-Induced VEGF Levels in NSCLC Cells Are Modulated by PKA, Hyaluronan, and p53
Source: Int J Mol Sci. 2025 Nov 17;26(22):11103. doi: 10.3390/ijms262211103 (PMC12652776; doi:10.3390/ijms262211103)
Supplement: Supplementary file 1 [file ijms-26-11103-s001.zip › ijms-3926130-supplementary.pdf]

Manuscript ID: ijms-3926130

Title: Nicotine-Induced VEGF Levels in NSCLC Cells Are Modulated by PKA, Hyaluronan, and p53

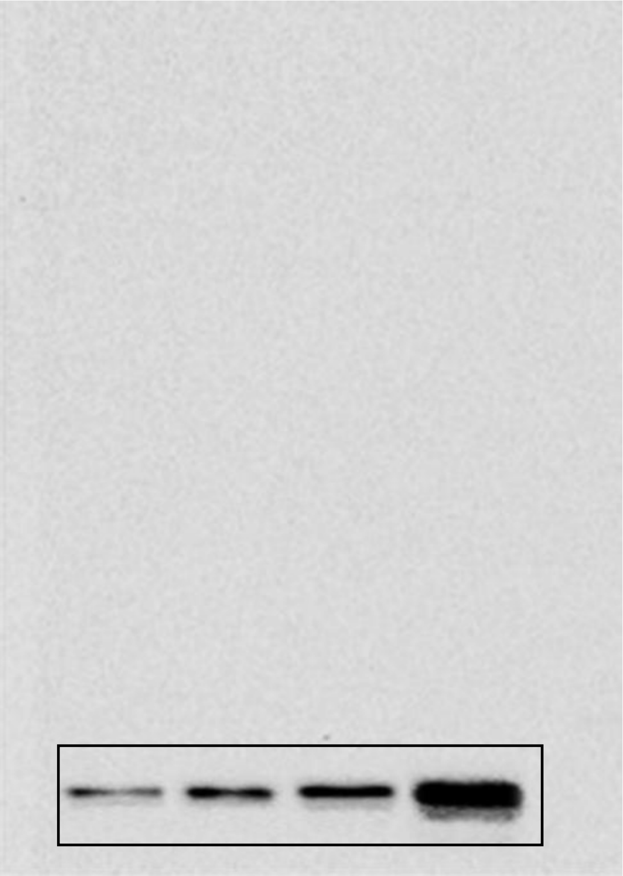

The image above was cropped to produce the image below.

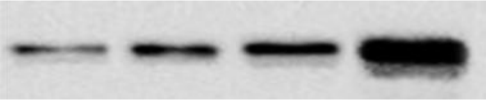

Shown on Figure 1I Top

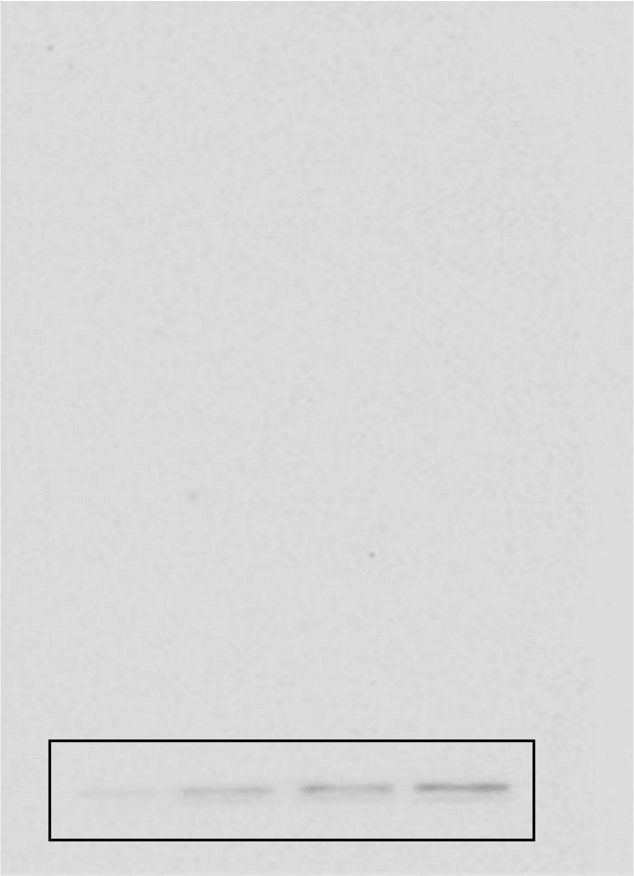

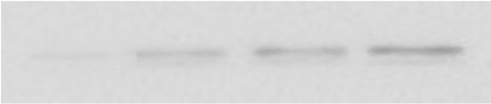

The image above was cropped to produce the image below.

Shown on Figure 1I Middle

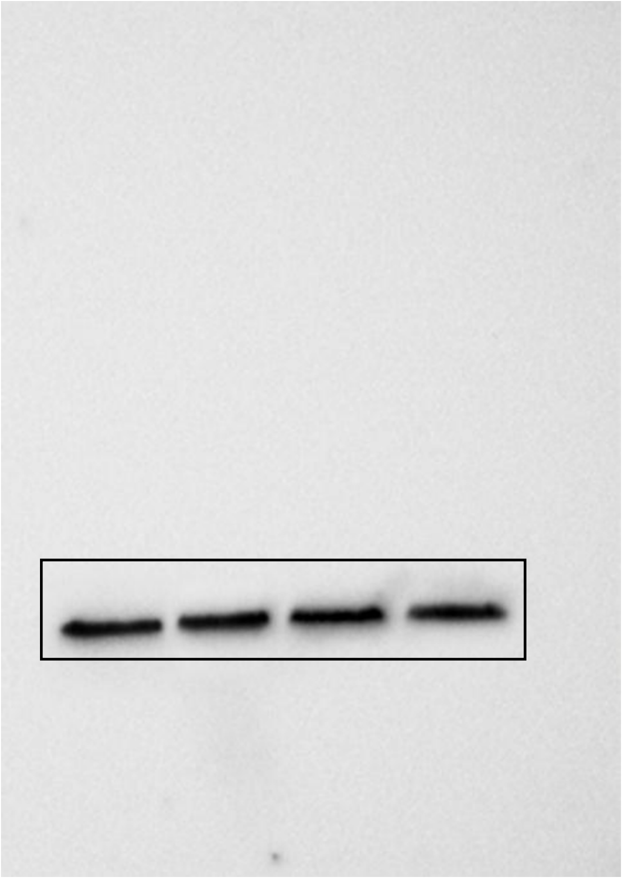

The image above was cropped to produce the image below.

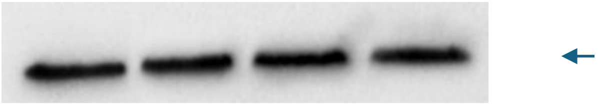

Shown on Figure 1I Bottom

I

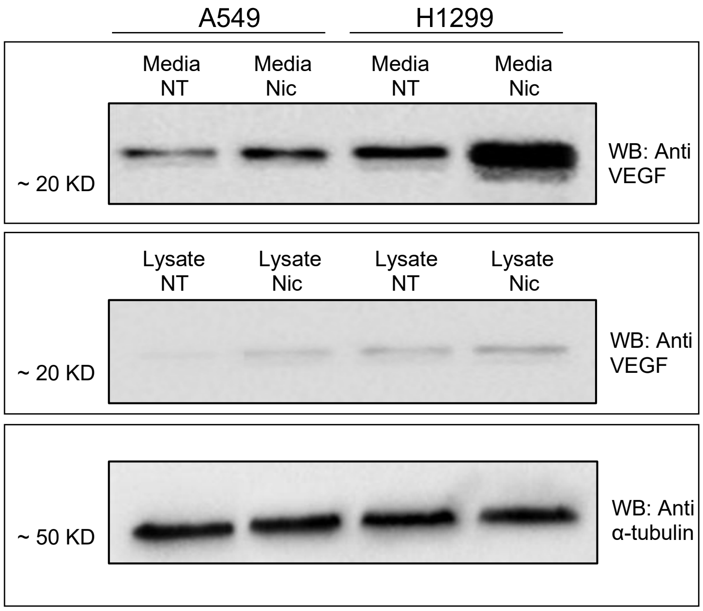

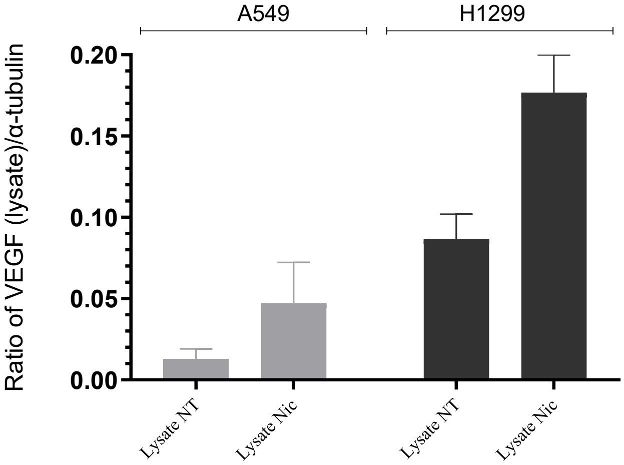

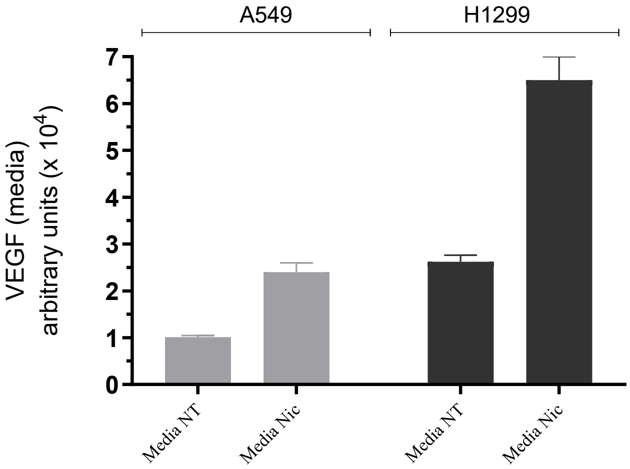

Western blotting using the indicated antibodies was carried out on the same concentration of total protein. Western blot band intensities were quantified by densitometry using ImageJ (NIH), with background subtraction.

Antibodies used

1) Human/primate mouse VEGF antibody (MAB293-100)
   https://www.rndsystems.com/products/human-primate-vegf-antibody-26503_mab293
2) α-tubulin mouse monoclonal antibody (DM1A)
   https://www.thermofisher.com/antibody/product/alpha-Tubulin-Antibody-clone-DM1A-Monoclonal/62204
3) Goat anti-mouse IgG (H+L) superclonal secondary antibody, HRP conjugate (A28177)
   https://www.thermofisher.com/antibody/product/Goat-anti-Mouse-IgG-H-L-Secondary-Antibody-Recombinant-Superclonal/A28177

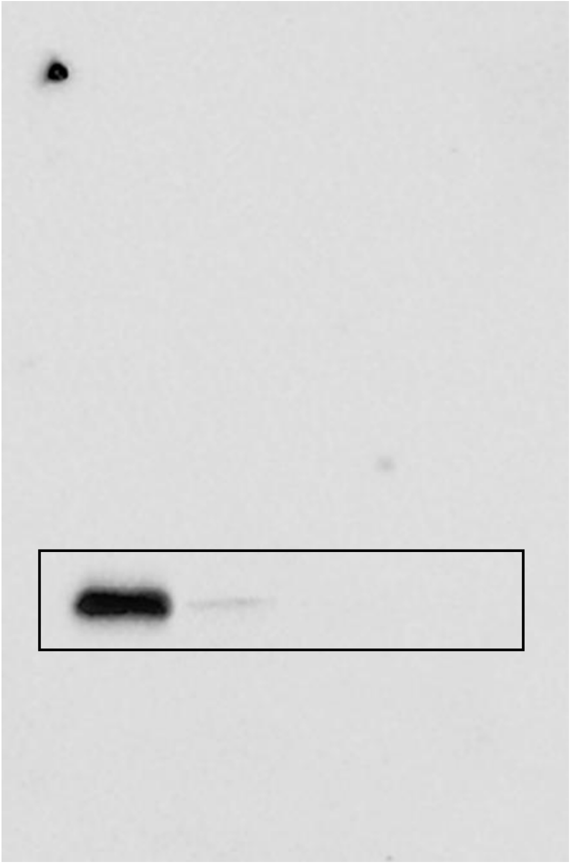

The image above was cropped to produce the image below.

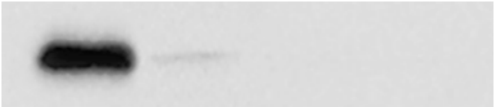

Shown on Figure 5A Top

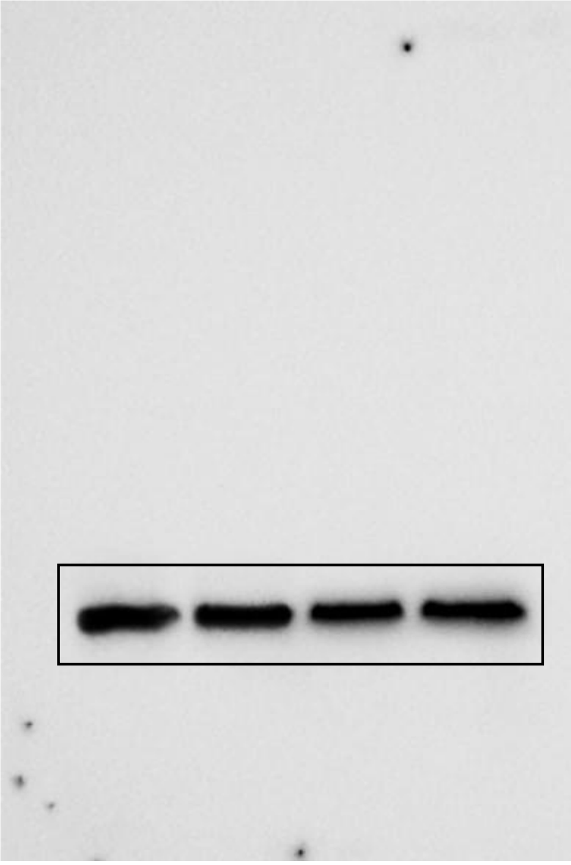

The image above was cropped to produce the image below.

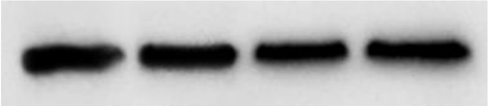

Shown on Figure 5A Bottom

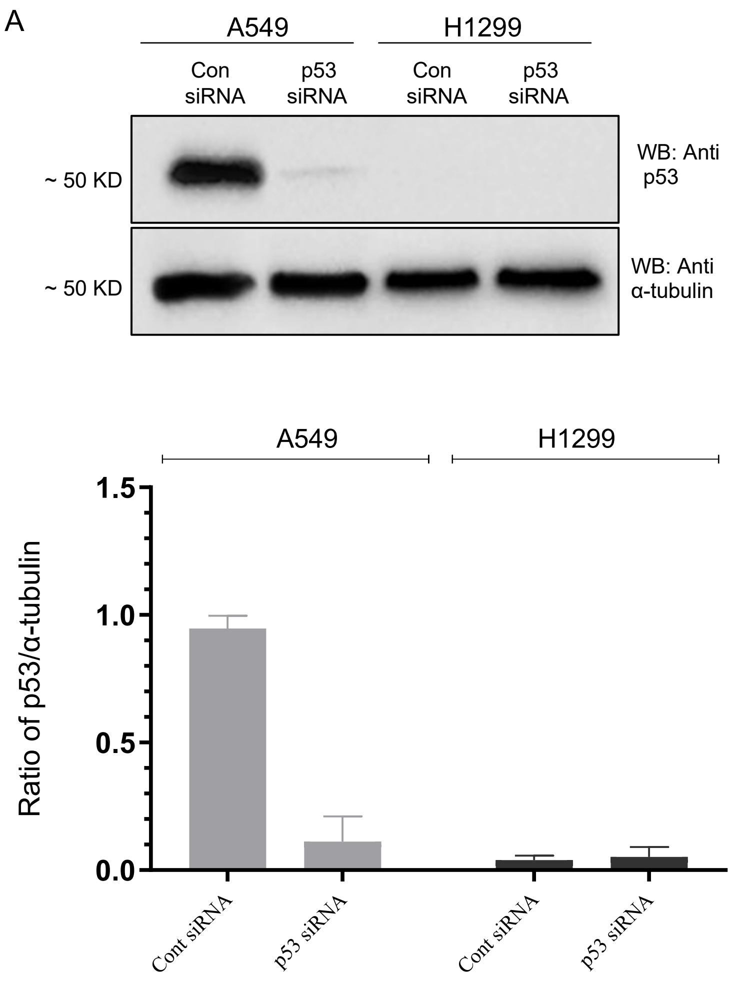

Western blotting using the indicated antibodies was carried out on the same concentration of total protein. Western blot band intensities were quantified by densitometry using ImageJ (NIH), with background subtraction.

Antibodies used

1) p53 rabbit antibody (9282) https://www.cellsignal.com/products/primary-antibodies/p53-antibody/9282?srsltid=AfmBOooaA73B_peh0LqICP1SMBEzdWtTM1b81Gqs4ujSTDFSzXy1HsV6
2) Goat anti-rabbit IgG (H + L) secondary antibody (HRP, 31466)

https://www.thermofisher.com/antibody/product/Goat-anti-Rabbit-IgG-H-L-Secondary-Antibody-Polyclonal/31466

3) α-tubulin mouse monoclonal antibody (DM1A)
https://www.thermofisher.com/antibody/product/alpha-Tubulin-Antibody-clone-DM1A-Monoclonal/62204
4) Goat anti-mouse IgG (H+L) superclonal secondary antibody, HRP conjugate (A28177)
https://www.thermofisher.com/antibody/product/Goat-anti-Mouse-IgG-H-L-Secondary-Antibody-Recombinant-Superclonal/A28177

siRNA used

1) SignalSilence p53 siRNA I (6231)
https://www.cellsignal.com/products/sirna/p53-sirna-i/6231?srsltid=AfmBOor_6_7FB6pxWSs3kxpeyykhR8HVL5aBunJfM1MtYUKcVIX5Ctwg
2) SignalSilence control siRNA (Unconjugated, 6568)
https://www.cellsignal.com/products/sirna/control-sirna-unconjugated/6568?srsltid=AfmBOoonaLfPFXMTqU1W2muBGCvSz6NKeN6Qagfdm_xztbcRZbkB0auj
